# Loose Particle Material Identification for Sealed Electronic Devices Using Pulse Endpoint Detection and CEEMDAN Feature Optimization

**DOI:** 10.3390/s26185875

**Published:** 2026-09-16

**Authors:** Zhichao Ren, Kunfeng Wang, Yongjian Lu, Shu Song, Xudong Zou

**Affiliations:** 1 School of Electronics and Integrated Circuits, Aerospace Information Technology University, Jinan 250132, China; renzc@aitech.edu.cn (Z.R.); luyj@aitech.edu.cn (Y.L.); songshu@aircas.ac.cn (S.S.); 2 State Key Laboratory of Transducer Technology, Aerospace Information Research Institute, Chinese Academy of Sciences, Beijing 100190, China; wangkunfeng@aircas.ac.cn

**Keywords:** loose particles, Particle Impact Noise Detection (PIND), CEEMDAN decomposition, feature extraction, BP neural network

## Abstract

Loose particles inside aerospace-sealed electronics cause circuit short circuits and contact faults. Particle Impact Noise Detection (PIND) relies on piezoelectric acoustic emission (AE) sensors to capture collision pulses, yet raw sensor signals are heavily contaminated by background noise, leading to severe time–frequency feature aliasing and low particle material recognition accuracy. This work proposes a sensing signal optimization method combining pulse endpoint detection and Complete Ensemble Empirical Mode Decomposition with Adaptive Noise (CEEMDAN) decomposition for PIND acoustic-sensing systems. First, a frequency-domain variance dual-threshold algorithm extracts valid collision pulses from noisy sensor output and eliminates invalid noise segments. Second, CEEMDAN reconstruction with kurtosis-based IMF screening suppresses high-frequency impulsive noise and low-frequency trend components, and seven-dimensional time–frequency-fused features are extracted for classification. A two-hidden-layer back-propagation (BP) neural network identifies four typical contaminants: copper particles, solder particles, rubber particles, and epoxy particles. Comparative tests against EMD, EEMD, and wavelet thresholding show that the proposed CEEMDAN-based method raises overall classification accuracy from 71.8% (no denoising) to 85.1%. This approach improves the discrimination performance of PIND acoustic-sensing platforms and supports aerospace-packaging defect tracing.

## 1. Introduction

With the rapid advancement of aerospace engineering, precision measurement and control, and high-end equipment industries, the integration density and precision of integrated circuits and various sealed electronic devices have continuously improved. Their operational stability and service reliability directly determine the safe operation of complete equipment. During manufacturing, packaging, assembly and long-term service, tiny loose particles such as metal debris, plastic residues and ceramic fragments are easily generated inside sealed electronic devices. Concealed inside the device cavity and undetectable via visual inspection, these internal contaminants may shift and collide under vibration and shock-operating conditions, triggering critical malfunctions including circuit short circuits, poor electrical contact and mechanical jamming. They constitute an important potential failure source for precision electronic equipment.

Particle Impact Noise Detection (PIND), the prevailing non-destructive testing technique for loose particle inspection in sealed devices, has been widely deployed for quality screening of integrated circuits and sealed electronic components owing to its high sensitivity, non-destructive nature and broad adaptability [1]. Distinct from simple binary detection of particle existence, material identification of loose particles enables precise discrimination of different contaminants. It serves as a fundamental basis for evaluating hazard levels of internal particles and predicting failure risks, and also provides vital technical support for tracing particle-generation sources, locating packaging-process defects, and targeted optimization of device production and encapsulation workflows [2]. Therefore, material recognition of loose particles carries prominent engineering significance for improving mass production quality and long-term reliability of precision electronic devices.

In recent decades, scholars worldwide have conducted extensive research on PIND signal analysis and loose particle material identification for sealed electronic devices, progressively introducing signal processing and machine-learning algorithms to this field and advancing detection technology from manual interpretation to intelligent classification. Chen et al. [3,4,5] first constructed an automatic PIND testing platform and subsequently proposed a particle material identification scheme combining PCA dimensionality reduction and SVM, which represents an early classic machine-learning framework in this research domain. Later, the same research team adopted Mel-Frequency Cepstral Coefficients (MFCCs) as core frequency-domain features and integrated Hidden Markov Models (HMMs) to implement particle classification [6]. Nevertheless, this method only leverages single-type features without prior signal denoising, resulting in limited anti-interference performance. Taking sealed relays as research objects, Li et al. analyzed PIND collision signals, screened multiple typical time-domain and frequency-domain features as identification indicators, adopted a multi-layer perceptron model with hyperparameter optimization, and significantly boosted particle classification accuracy by refining network architectures [7]. Wang et al. targeted aerospace power supply devices. They used stochastic resonance to amplify weak PIND signals and built an LVQ network for particle classification [8]. The method achieves over 90% detection accuracy and 80% material recognition accuracy, yet it only adopts limited features and lacks strong generalization for diverse particle samples. The above literature verifies the feasibility of recognition frameworks based on time–frequency features and machine learning; however, most existing methods directly extract features from raw PIND signals without deep denoising and decomposition of weak collision pulses, leading to limited feature distinguishability under low signal-to-noise ratio (SNR) conditions.

Complete Ensemble Empirical Mode Decomposition with Adaptive Noise (CEEMDAN) is a mainstream algorithm for processing nonlinear, non-stationary weak signals, which has been extensively applied in fault diagnosis and signal classification [9,10,11]. Traditional EMD and EEMD suffer from inherent drawbacks including mode mixing and residual noise, whereas CEEMDAN supports adaptive signal decomposition and demonstrates prominent superiority in processing weak acoustic emission and vibration signals [12]. Targeting low-SNR vibration and acoustic emission signals of rotating machinery, Li et al. [13] proposed a hybrid feature-extraction framework integrating CEEMDAN and multi-scale entropy. They screened valid intrinsic mode function (IMF) components by correlation coefficients and variance contribution rates to eliminate noise and spurious modes, constructed composite feature sets fused with time-domain, frequency-domain and nonlinear entropy indicators, and quantitatively compared the decomposition performance of EMD, EEMD and CEEMDAN via comparative experiments, thoroughly validating the advantages of CEEMDAN for weak signal decomposition. For industrial strong-noise scenarios, Liu et al. [14] applied CEEMDAN to preprocess bearing acoustic emission signals, realized multi-dimensional feature fusion and lightweight neural network-based fault classification, and further discussed feature redundancy issues, confirming that the technical route of “CEEMDAN denoising + multi-feature fusion” can substantially strengthen the classification robustness of models under complex working conditions. The technical frameworks of the two aforementioned studies are highly consistent with the core idea of this work, offering crucial references for PIND signal preprocessing and feature mining.

Comprehensive analysis of existing research reveals three prominent limitations in current PIND signal-based loose particle material identification systems for sealed electronic devices. First, most existing studies directly analyze complete raw PIND acquisition signals, which contain lengthy silent baseline segments without valid shock information. Direct CEEMDAN decomposition of such full-length signals incurs excessive iterative computation and extremely low processing efficiency; additionally, low-frequency trend components within long baselines easily induce mode mixing, and the combined interference of noise and baseline distortion leads to a severe overlap of feature distributions across different particle materials. Second, feature construction forms are overly simplistic, relying solely on basic time-domain or frequency-domain metrics, while collaborative fusion schemes integrating time-domain, frequency-domain and nonlinear entropy multi-dimensional indicators are insufficiently exploited. Third, the front-end signal purification preprocessing module is decoupled from back-end denoising and feature optimization procedures, resulting in poor coupling of the preprocessing pipeline and failure to fully exploit the classification potential of subsequent machine-learning models.

To address these limitations, we propose a loose particle material identification method for sealed electronic devices based on pulse endpoint detection and CEEMDAN-based feature optimization. First, a frequency-domain variance dual-threshold endpoint detection algorithm locates and extracts valid collision pulses from raw sensor signals, removing silent baseline segments while preserving the pulse waveform. This step reduces the data length input to CEEMDAN and avoids mode mixing caused by long baseline segments. Second, the purified pulse signal is decomposed by CEEMDAN, and IMF components are selected by kurtosis. Specifically, IMF3–IMF7 are retained for signal reconstruction, while IMF1–IMF2 (impulsive noise) and IMF8 (low-frequency trend) are discarded. Seven features covering time-domain, frequency-domain and nonlinear entropy metrics are then extracted from the reconstructed signal. Finally, a two-hidden-layer BP neural network classifies four types of loose particles: copper, solder, rubber, and epoxy. A leave-one-mass-gradient-out protocol is used: 1200 samples from three mass gradients form the training set, and 400 samples from an unseen mass gradient form the test set. Each experiment is repeated four times, and results are reported as mean ± standard deviation. Three conventional denoising methods—EMD, EEMD, and wavelet thresholding—are included as benchmarks for comparison. Experiments on measured signals show that pulse preprocessing reduces CEEMDAN computation time and suppresses mode mixing. The proposed CEEMDAN-based method achieves an overall classification accuracy of 85.1%, outperforming the no-denoising control (71.8%), EMD (74.5%), wavelet thresholding (76.0%), and EEMD (76.8%). The combined pipeline reduces feature overlap between particle materials and improves classification accuracy compared with using raw signals directly. The proposed method provides a practical framework for reliability inspection of sealed electronic components and traceability of packaging process defects.

## 2. Related Works

### 2.1. Loose Particle Noise Detection System

**Figure 1:**
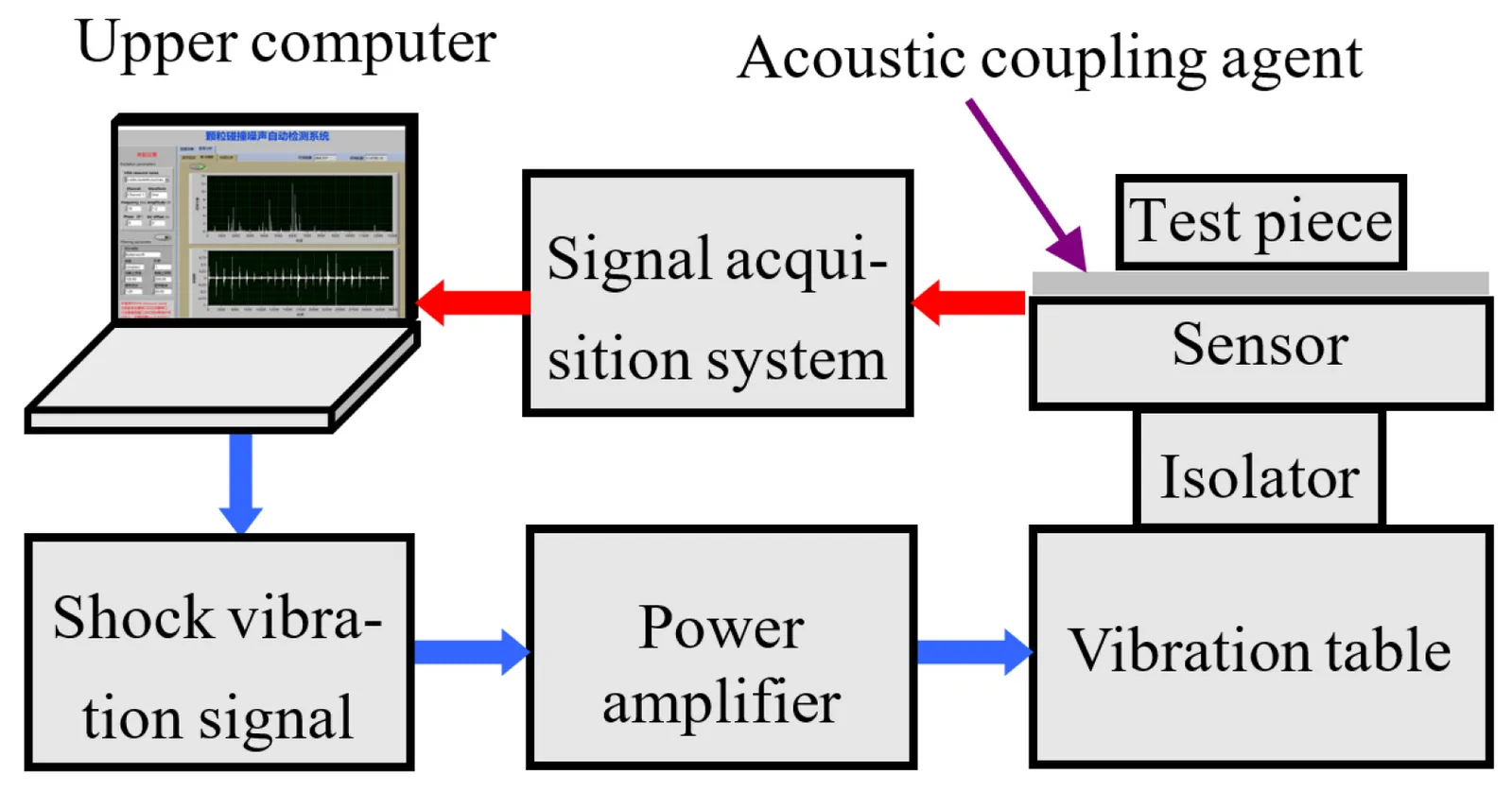
Schematic diagram of the PIND testing principle.

Particle Impact Noise Detection (PIND) serves as the standard non-destructive testing technique for loose contaminants within sealed electronic devices. The schematic of its operating principle is presented in Figure 1.

**Figure 2:**
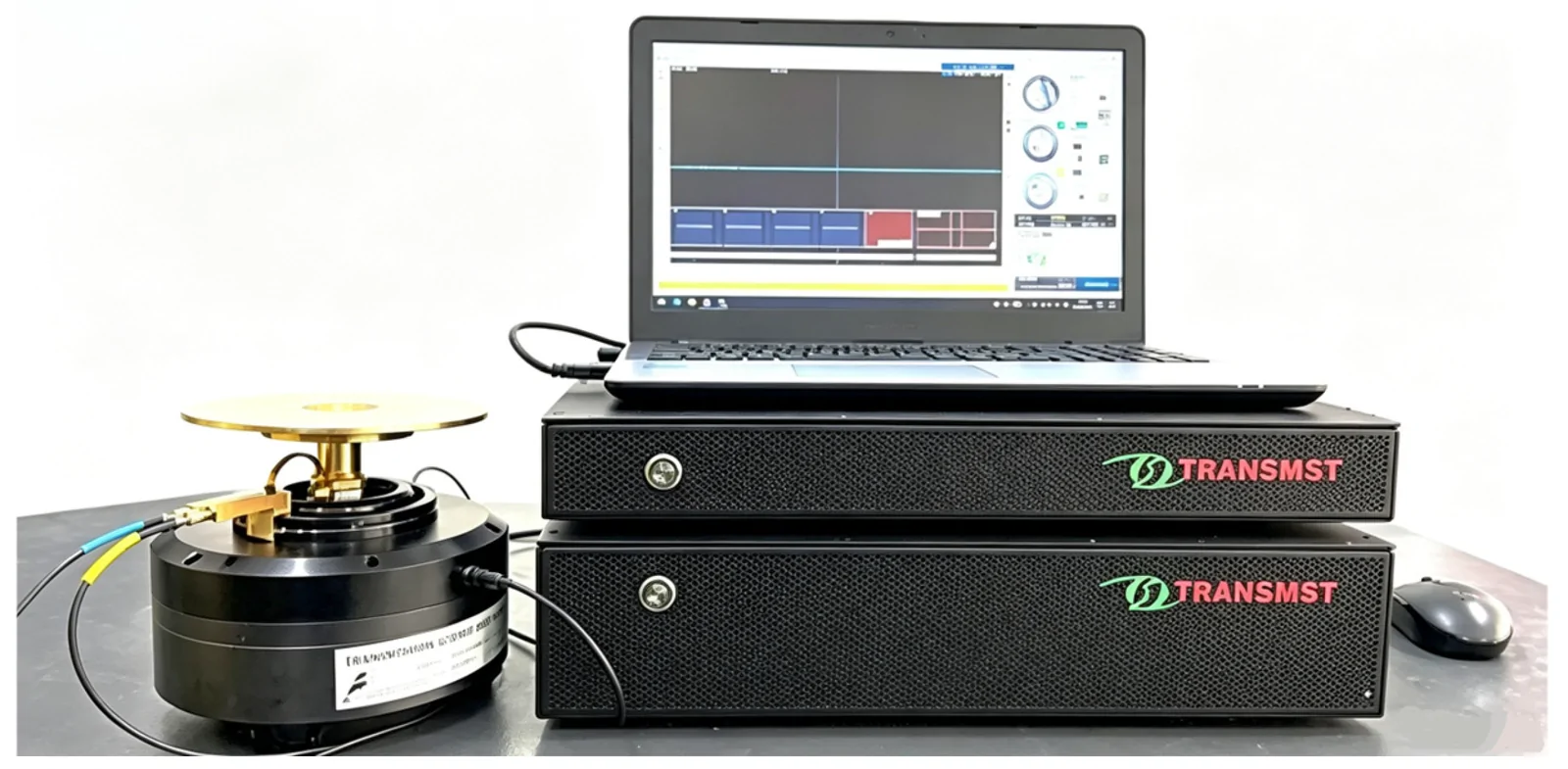
Photograph of the experimental PIND test platform.

Tiny loose particles are confined inside the cavity of sealed electronic components. Under normal static conditions, surface adhesion forces attach these lightweight particles to inner walls or internal structures, keeping them immobile. In the PIND test procedure, impact excitation is first exerted to detach particles from adsorbed surfaces. The device is then vibrated at a designated frequency, which drives loose particles to collide randomly with internal walls and components and generate faint acoustic signals [15]. A resonant acoustic emission (AE) sensor captures these collision signals, which are transmitted to a host computer through a high-speed data acquisition (DAQ) card. Further signal analysis and processing allow judges to identify the existence of loose particles inside the Device Under Test (DUT). The real-world experimental setup is shown in Figure 2.

The experimental platform adopts the WSD-DOL50 desktop PIND tester manufactured by Nanjing Maergou Transmission Technology Co. Ltd. (Nanjing, China), which is specially customized for testing miniature sealed electronic devices. A resonant piezoelectric AE sensor with a central frequency of 150 kHz and a bandwidth of 50–400 kHz is used to capture collision signals. The signals are sampled at 1 MHz with 16-bit resolution, and a preamplifier with 40 dB gain is applied. A 20–500 kHz band-pass filter is used during acquisition. This integrated system equips piezoelectric acoustic sensors and high-precision DAQ modules. These excitation parameters are optimized through pre-tests as described in Section 2.2: impact acceleration of 200 g, vibration frequency of 30 Hz, and vibration acceleration of 5 g, enabling stable acquisition of collision signals generated by internal loose particles.

### 2.2. Standardized PIND Test Environment

Particle Impact Noise Detection (PIND) technology is widely used for sealed electronic components with enclosed cavities, such as sealed relays, switches, oscillators and integrated circuit modules. Finished components of different models differ greatly in cavity geometry. Direct adoption of commercial products for experiments will introduce inconsistent variables, which prevents the construction of a standardized test environment and hinders batch sample preparation and repeatable verification.

**Figure 3:**
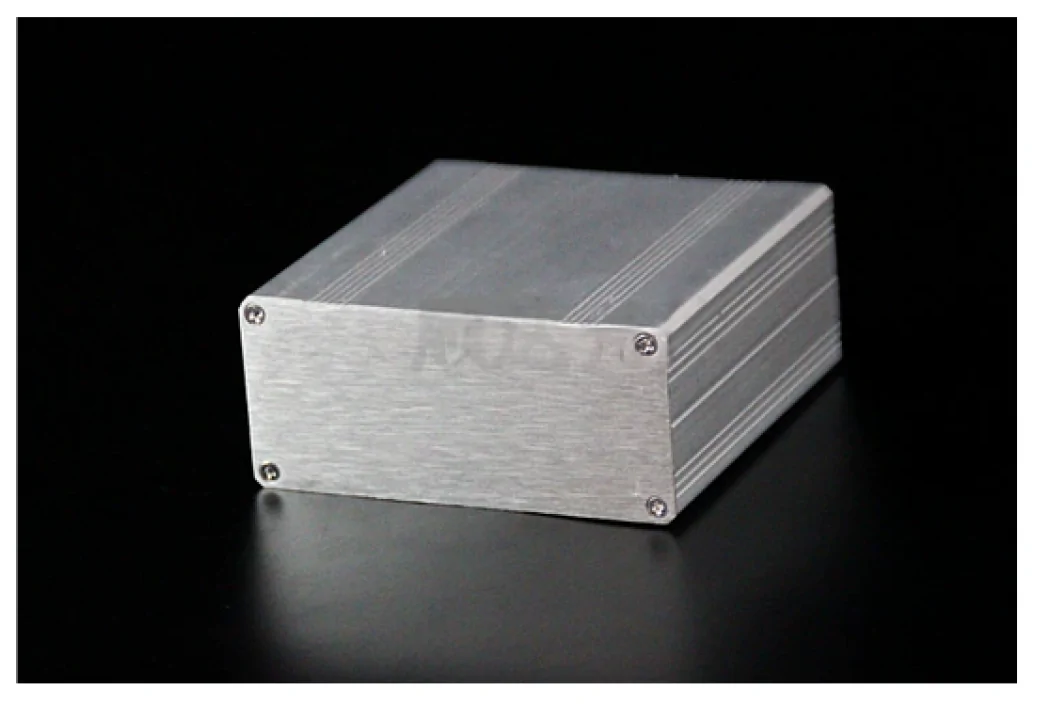
Photograph of the machined aluminum-alloy-simulated cavity.

To eliminate this limitation, precision machining is adopted in this work to fabricate standardized aluminum-alloy (6061)-simulated cavities as substitutes for real devices. The metal cavity provides acoustic impedance and damping characteristics representative of actual sealed electronic device packages, reducing the gap between simulated tests and real-world applications. The cavity measures 20 mm × 20 mm × 10 mm (length × width × height) with a wall thickness of 1 mm. The standardized aluminum cavity ensures consistent boundary conditions across all experiments, forming a stable, controllable test carrier for capturing collision signals of loose particles. A photograph of the machined aluminum cavity is shown in Figure 3. All experiments are conducted at room temperature under ordinary laboratory ambient noise.

All excitation parameters are configured within the range specified by the Chinese military standard GJB 65B [16] Methods for Particle Impact Noise Detection of Electronic Components, which is consistent with equivalent international standards such as MIL-STD-883L-2019 [17] (United States) and IEC 60749-16 [18] (International Electrotechnical Commission). A series of pre-tests are carried out on the customized aluminum cavity to optimize vibration excitation parameters. The results demonstrate that a vibration frequency of 30 Hz yields the most steady collisions between loose particles and cavity walls. Accordingly, 30 Hz is adopted as the standard vibration frequency throughout all formal tests, with a vibration acceleration of 5 g and an impact acceleration of 200 g applied prior to each test.

### 2.3. Experimental Design

To build a complete dataset for material identification, four types of typical loose particles generated during the manufacturing and packaging of sealed electronic devices are selected as experimental samples: copper particles, solder particles, rubber particles, and epoxy particles. The detailed experimental scheme is described as follows:

Sample preparation: Four mass gradients are defined within 0.10 mg–0.82 mg. To mitigate the confounding effect of particle mass on material classification, specimens of the four materials are prepared with closely matched masses at each gradient, as summarized in Table 1.

Repeated testing: A single loose particle specimen is placed inside the aluminum-alloy-simulated cavity, which is fixed on the PIND experimental platform for vibration testing at 30 Hz. Each specimen of every material and each mass gradient is measured 100 times repeatedly.

Signal acquisition and storage: During each test, acoustic emission signals from particle collisions are acquired in real time. The raw signals are conditioned and time-synchronized, then stored on the host computer for subsequent processing.

Dataset partition: For model training and evaluation, a leave-one-mass-gradient-out strategy is adopted. Samples at 0.1 mg, 0.2 mg and 0.8 mg form the training set (1200 samples in total, 100 samples per material per gradient), while samples at 0.4 mg are reserved as an independent test set (400 samples, 100 per material). The 0.4 mg gradient is completely unseen during training to evaluate the model’s generalization to new particle masses.

Experimental groups: Five experimental groups are configured with identical network architectures and hyperparameters, differing only in the signal preprocessing scheme: a control group with features extracted directly from concatenated pulses (no denoising), three conventional denoising groups using EMD, EEMD, and wavelet thresholding, and an experimental group using CEEMDAN-reconstructed signals. Each group is trained and tested four times with random network initializations, and classification results are reported as mean accuracy ± standard deviation.

## 3. Methods

The signal processing procedure in this study consists of three sequential stages. First, pulse extraction and concatenation are performed on the preprocessed loose particle signals to reduce data volume while improving the signal-to-noise ratio. Next, feature extraction is carried out to capture the material-related information contained in the collision signals. Finally, the extracted features are fed into a neural network to determine the presence of loose particles and identify their material properties.

### 3.1. Preprocessing of Loose Particle Signals

**Figure 4:**
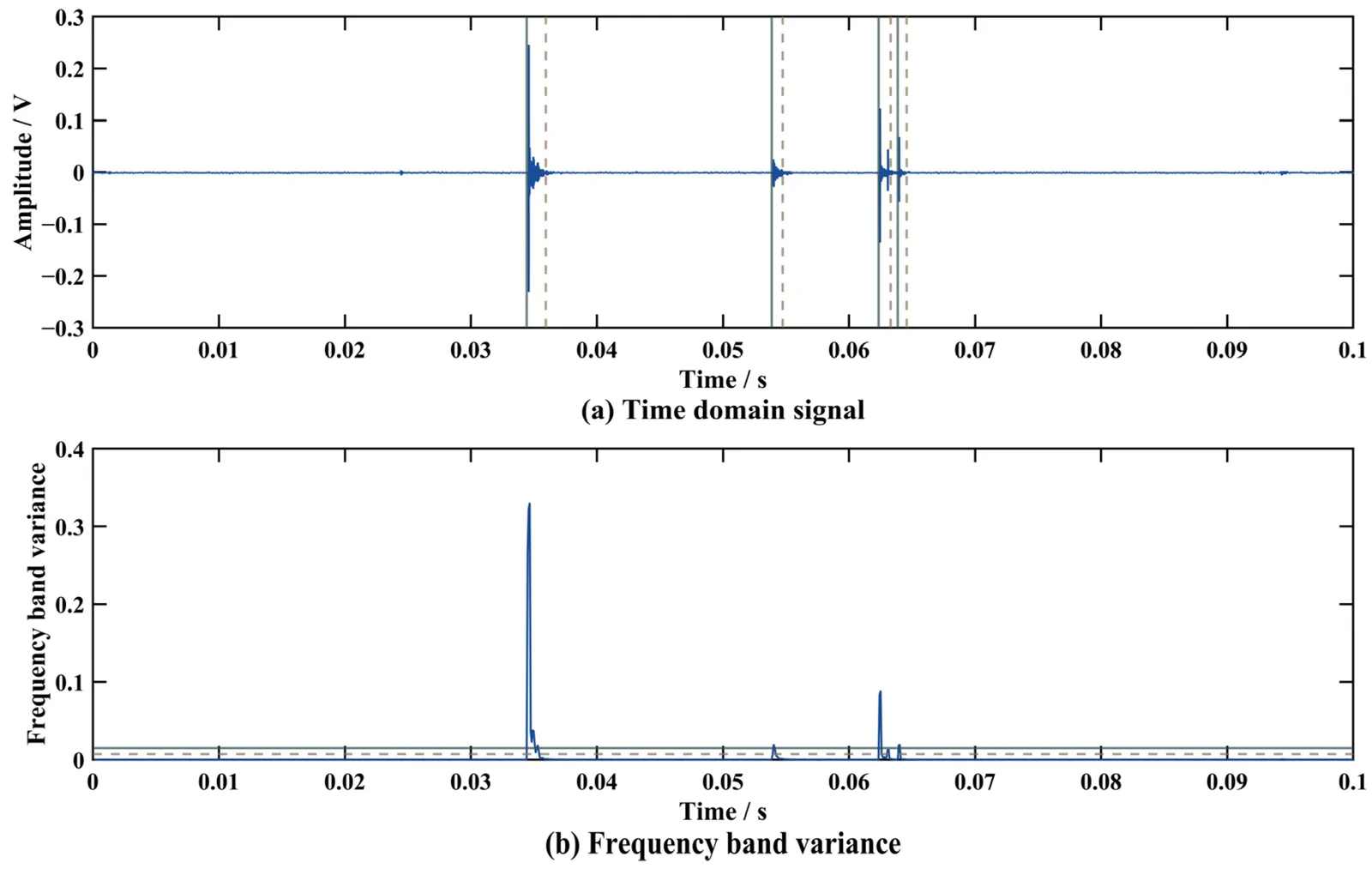
Pulse extraction results based on frequency-domain variance dual-threshold endpoint detection. The gray-green solid line represents threshold *T_i_*, and the yellow dashed line denotes threshold *T_o_*.

Raw PIND signals contain long static segments in addition to the collision pulses of interest. Direct time–frequency analysis without preprocessing introduces redundant computation and baseline interference. Chen et al. [19] proposed a short-term energy threshold method for pulse extraction. Building on this work, we use a frequency-domain variance dual-threshold method with a minimum pulse duration constraint to locate the start and end points of each collision pulse, as shown in Figure 4.

Specifically, a higher pulse admission threshold *T_i_* is defined on the short-time–frequency band variance to preliminarily locate potential events. A lower pulse termination threshold *T_o_* is derived from the average band variance *T_mean_*. Under the constraint of the minimum valid pulse duration *h*, the intersection points between the short-time frequency–band variance envelope and the threshold *T_o_* are identified to determine the boundaries of each collision pulse. The choice of these thresholds is closely tied to the characteristics of environmental noise and loose particle signals. Through extensive calibration experiments, the thresholds are set to *T_i_* = 3 *T_mean_* and *T_o_* = 1.5 *T_mean_*. This configuration enables reliable pulse extraction, followed by secondary stitching of the isolated segments, which significantly reduces the volume of raw data while preserving the key information required for subsequent analysis.

**Figure 5:**
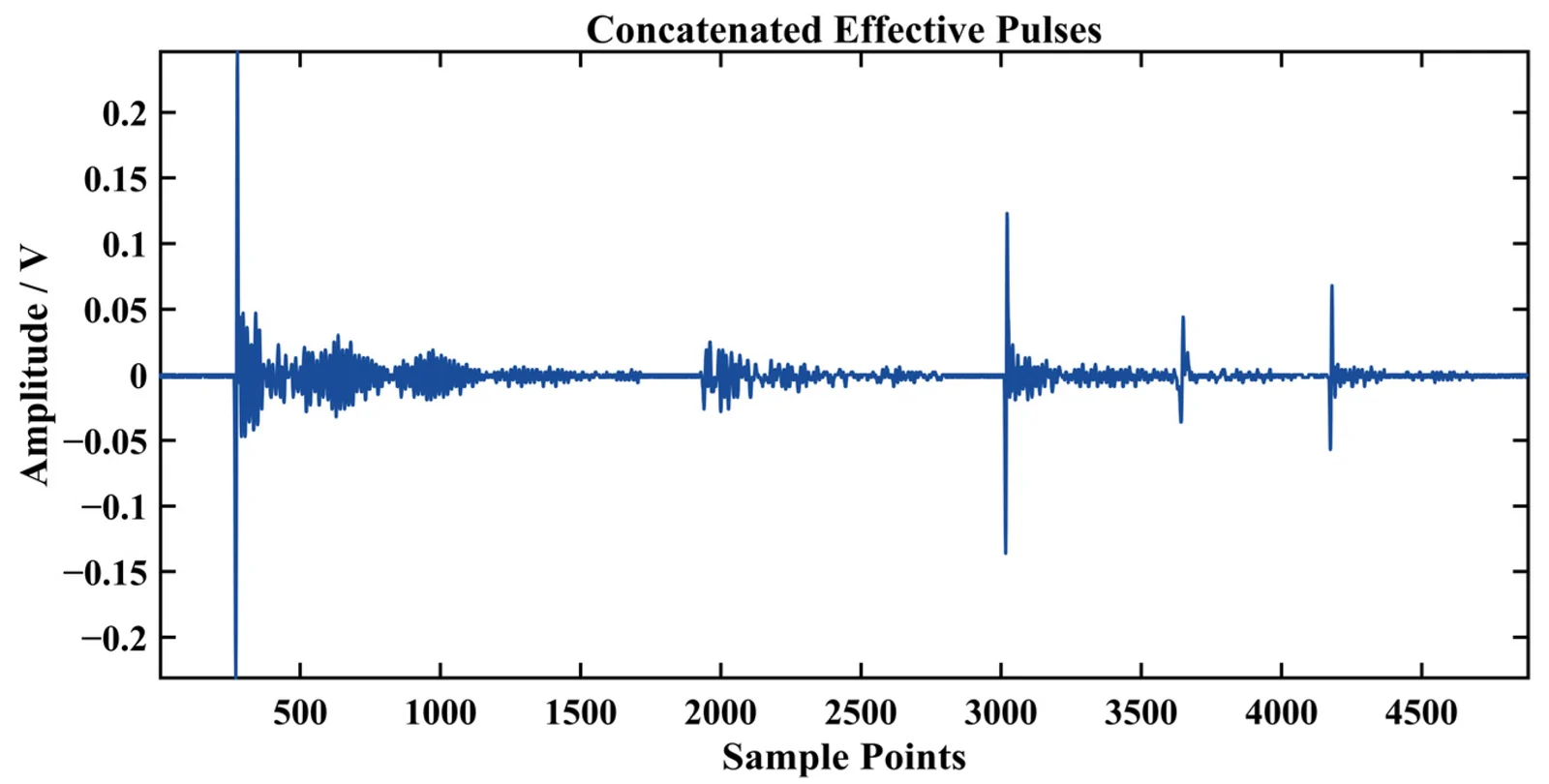
Waveform of concatenated effective collision pulses.

This concatenation step removes baseline noise intervals between pulses while preserving the amplitude and waveform of each collision, as shown in Figure 5. Without pulse extraction, CEEMDAN decomposition of a single raw acquisition requires 40–45 min. After pulse extraction and concatenation, the same decomposition takes only 5–7 min, reducing computation time by approximately 85%. The shortened input also avoids mode mixing caused by long baseline segments.

### 3.2. Signal Denoising

Effective denoising is essential for extracting reliable features from noisy PIND collision pulses. This section introduces the denoising methods used in this study. Three conventional methods—EMD, EEMD, and wavelet thresholding—are first described as benchmarks, followed by the proposed CEEMDAN method combined with kurtosis-based IMF screening.

#### 3.2.1. Principles of Conventional Denoising Methods

To evaluate the performance of the proposed CEEMDAN-based denoising scheme, three conventional denoising methods are selected as benchmarks: empirical mode decomposition (EMD), ensemble empirical mode decomposition (EEMD), and wavelet threshold denoising.

EMD. EMD is an adaptive decomposition method for nonlinear and nonstationary signals, which decomposes a signal into a set of intrinsic mode functions (IMFs) through an iterative sifting process. However, EMD suffers from mode mixing, where a single IMF may contain components of different scales, or components of the same scale may be distributed across multiple IMFs. In this study, EMD is applied to the concatenated pulse signals with a maximum of 5000 sifting iterations, and the resulting IMFs are screened using the same kurtosis-based criterion (threshold of 50) as the CEEMDAN method to ensure a fair comparison.

EEMD. EEMD was developed to alleviate the mode-mixing problem in EMD by repeatedly adding Gaussian white noise to the signal and averaging the resulting IMFs. The added white noise populates the time–frequency space uniformly, providing a reference scale for the sifting process. However, EEMD introduces residual noise in the reconstructed signal and may suffer from incomplete decomposition due to inconsistent IMF numbers across different noise-adding trials. In this study, EEMD is performed with 200 ensemble trials and a noise amplitude coefficient of 0.15, consistent with the CEEMDAN parameters, and the resulting IMFs are screened using the same kurtosis threshold of 50.

Wavelet threshold denoising. This method decomposes a signal into approximation and detail coefficients at multiple scales via discrete wavelet transform, applies a threshold to the high-frequency detail coefficients where noise predominantly resides, and reconstructs the signal from the modified coefficients. In this study, the Daubechies 4 (db4) wavelet is selected as the mother wavelet due to its orthogonality and compact support, the decomposition level is set to 5, the universal threshold (sqtwolog) is used for threshold estimation, and soft thresholding is applied to the detail coefficients.

For all three benchmark methods, the denoised signals are used to extract the identical seven-dimensional feature set described in Section 3.3, and classification is performed with the same BP neural network configuration. This design ensures that any performance difference can be attributed solely to the denoising method.

#### 3.2.2. CEEMDAN Decomposition and Kurtosis-Based Screening

Complete Ensemble Empirical Mode Decomposition with Adaptive Noise (CEEMDAN) is an adaptive decomposition method for nonlinear and nonstationary signals, developed from EMD and EEMD [20]. Conventional EMD suffers from mode mixing. EEMD reduces mode mixing by repeatedly adding Gaussian white noise, but introduces residual noise and incomplete decomposition. CEEMDAN adds adaptive noise at each decomposition stage and averages the resulting intrinsic mode functions (IMFs), which suppresses mode mixing and reduces residual noise while maintaining decomposition completeness. CEEMDAN is therefore suitable for processing weak collision signals acquired in PIND tests.

Let the raw discrete collision signal be *x*(*n*). The detailed decomposition procedures of CEEMDAN are illustrated as follows:

**Step1:** A set of adaptive Gaussian white noise *w^i^*(*n*) with distinct amplitudes is superimposed onto the raw signal to construct composite signals:

(1) $${\;x}^{i}\left(n\right)=\;x\left(n\right)+\;\varepsilon \omega^{i}\left(n\right),\;i=1,2,\ldots ,I$$

where *I* denotes the total number of noise-adding trials, and ε represents the noise amplitude coefficient.

**Step2:** EMD is performed on each composite signal to extract the first-order IMF component $imf_{1}^{i}(n)$. The ensemble average of all obtained $imf_{1}^{i}(n)$ is calculated to yield the first intrinsic mode component of CEEMDAN decomposition:

(2) $$\bar{imf_{1}}\left(n\right)=\frac{1}{I}\sum_{i=1}^{I}imf_{1}^{i}\left(n\right)$$

**Step3:** The first residual component is computed as:

(3) $$r_{1}\left(n\right)=x\left(n\right)\;-\;\bar{imf_{1}}\left(n\right)$$

Treat $r_{1}(n)$ as the new signal to be decomposed, and repeat the above steps to sequentially solve each order of IMF components.

**Step4:** Iterate the decomposition process continuously until the final residual component $r_{k}(n)$ presents a monotonic trend and cannot be further decomposed.

Ultimately, the raw signal can be decomposed into the superposition of multiple IMFs and the residual term:

(4) $$x\left(n\right)=\sum_{j=1}^{k}\;\bar{imf_{j}}\left(n\right)+r_{k}(n)$$

where $\bar{imf_{j}}\left(n\right)$ is the *j*-th intrinsic mode function, and $r_{k}(n)$ stands for the final residual component. Each IMF corresponds to a different frequency band, ranging from high-frequency oscillations in the early IMFs to low-frequency trends in the later IMFs. In this work, the CEEMDAN parameters are set as follows: noise amplitude coefficient ε=0.15, total ensemble trials I=200, and maximum iteration number of 5000.

**Figure 6:**
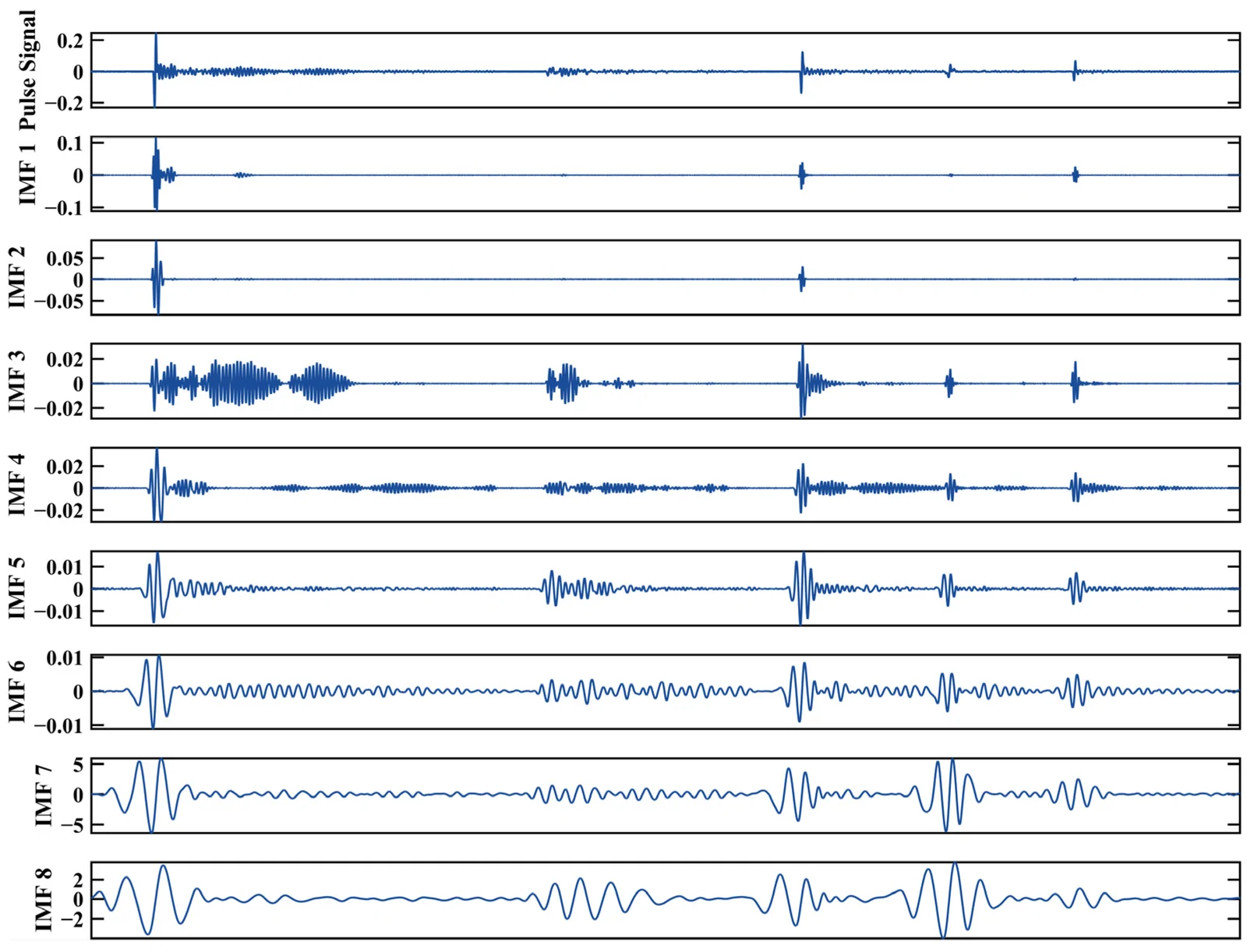
CEEMDAN decomposition results of concatenated pulse signal.

After decomposition, not all IMFs contain valid collision information. Figure 6 shows the CEEMDAN decomposition result of a concatenated pulse signal, including the original signal and eight IMF components. Kurtosis is used to distinguish valid components from noise-dominated components.

Kurtosis is defined as the fourth standardized moment of a signal, which quantifies the sharpness of the amplitude distribution. A Gaussian signal has a kurtosis of 3, while signals with impulsive spikes exhibit kurtosis values well above 3. For PIND signals, high-frequency IMFs (IMF1–IMF2) consist of sparse, sharp spikes induced by random background noise, and therefore show extremely high kurtosis. In contrast, IMFs carrying valid collision pulses (IMF3–IMF7) exhibit sustained oscillatory structures with moderate kurtosis, while the lowest-frequency IMF (IMF8) represents a slow-varying trend with the lowest kurtosis. Unlike energy-based or correlation-based criteria, which are affected by the large amplitude of noise spikes, kurtosis directly captures the impulsiveness difference between noise-dominated and signal-dominated components, making it suitable for IMF screening in PIND applications.

The kurtosis of each IMF is calculated, and a threshold of 50 is applied. IMFs with kurtosis above 50 (IMF1 and IMF2, dominated by impulsive noise) are discarded. IMFs with kurtosis below 50 (IMF3–IMF7, containing collision pulse structures) are retained. IMF8 is a low-frequency trend term and is also excluded. The kurtosis values of all eight IMFs are listed in Table 2. The retained IMFs (IMF3–IMF7) are linearly superimposed to reconstruct the denoised signal, which preserves collision information for material identification while suppressing impulsive noise and low-frequency drift.

### 3.3. Signal Feature Extraction

**Figure 7:**
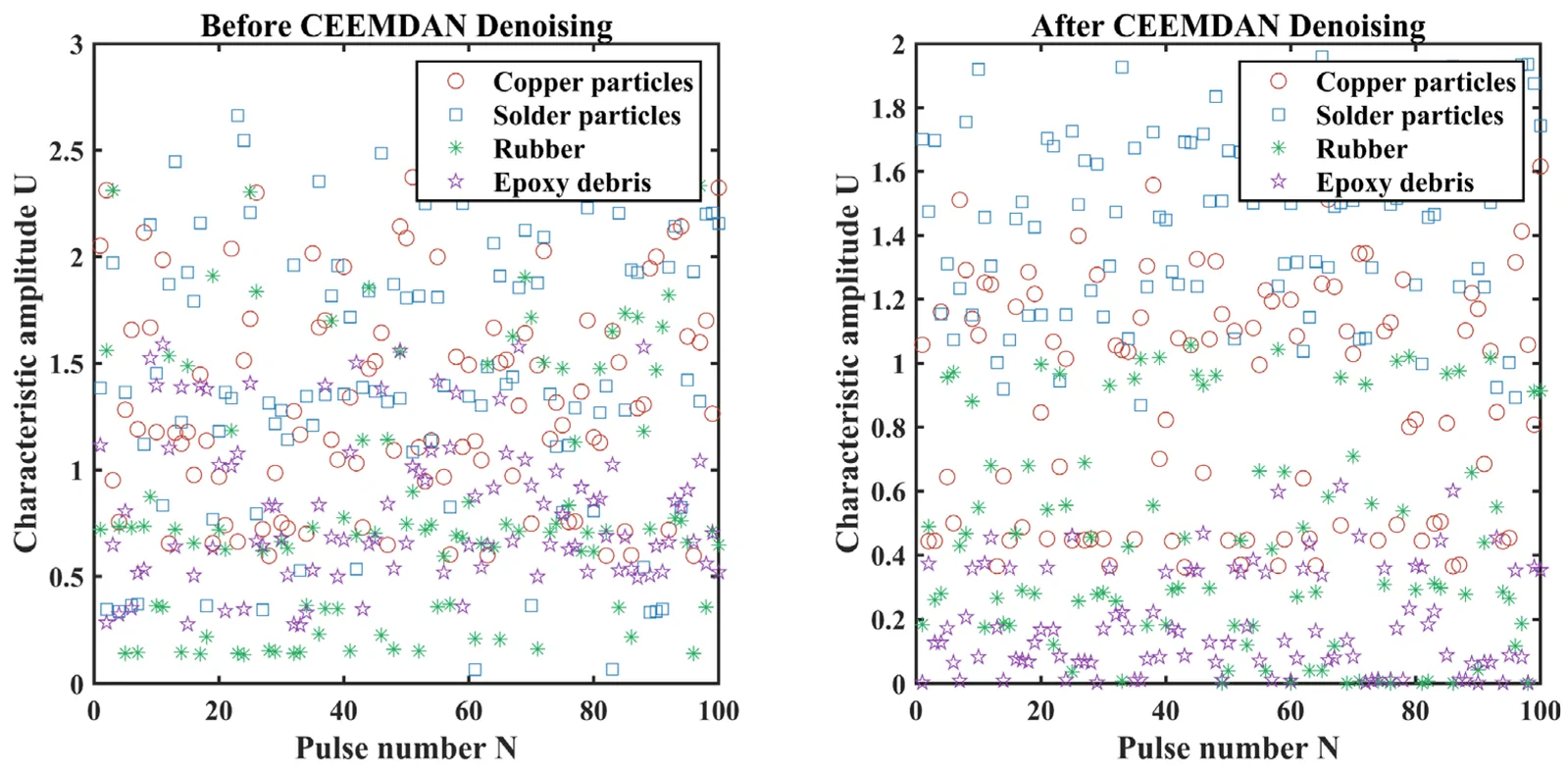
Time-domain energy before and after CEEMDAN denoising.

**Figure 8:**
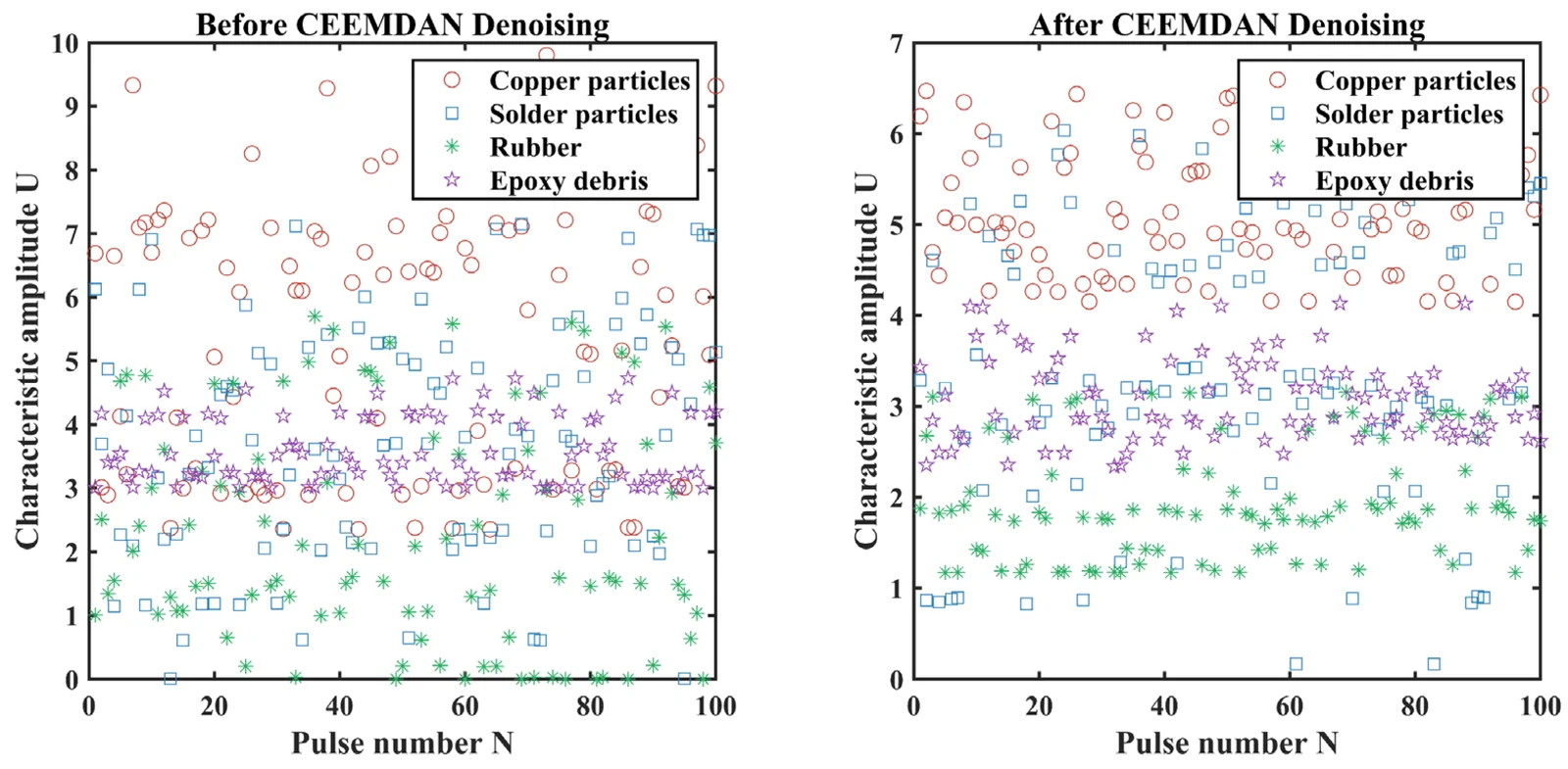
Spectrum variance before and after CEEMDAN denoising.

**Figure 9:**
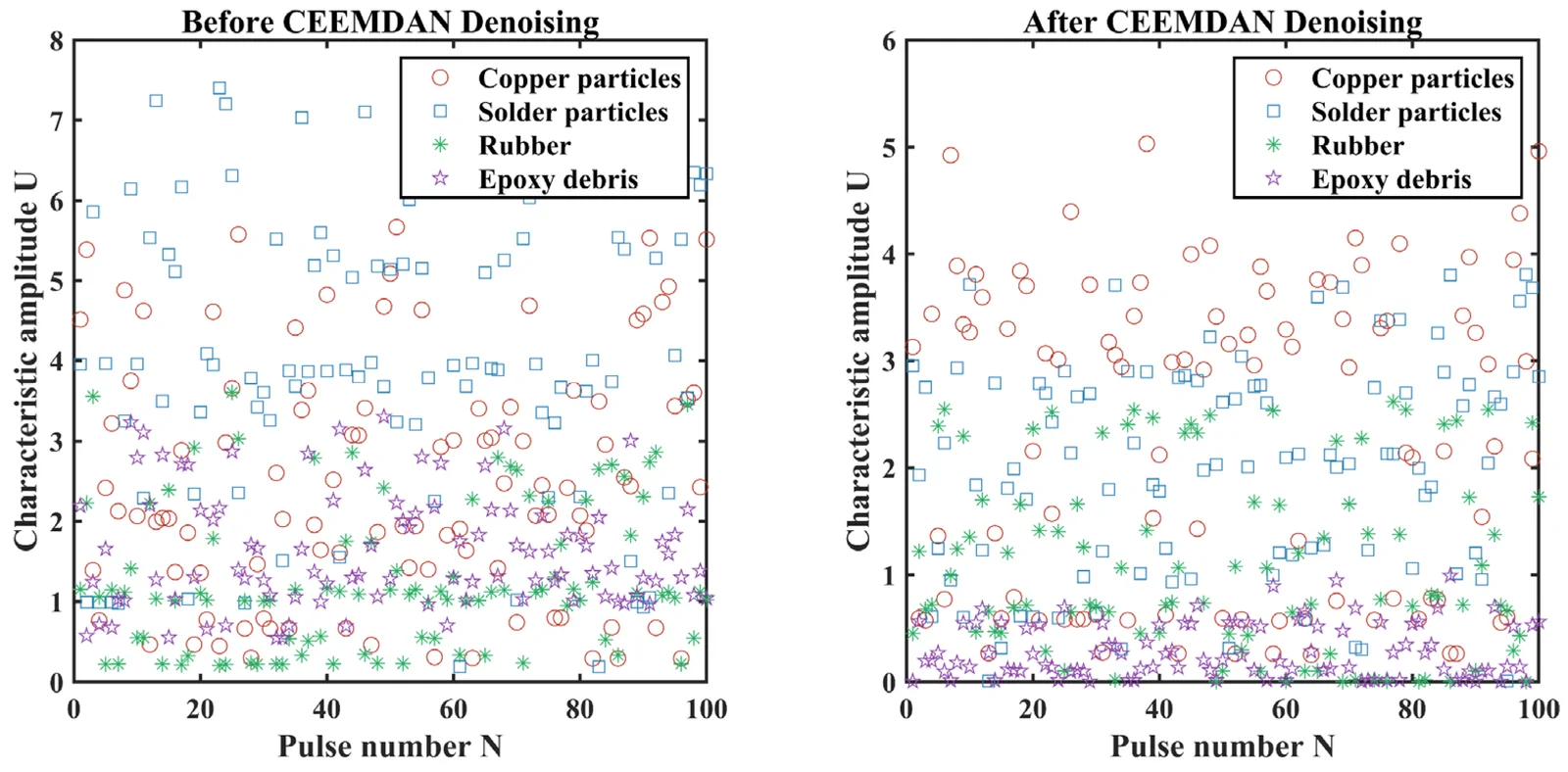
Autocorrelation peak before and after CEEMDAN denoising.

**Figure 10:**
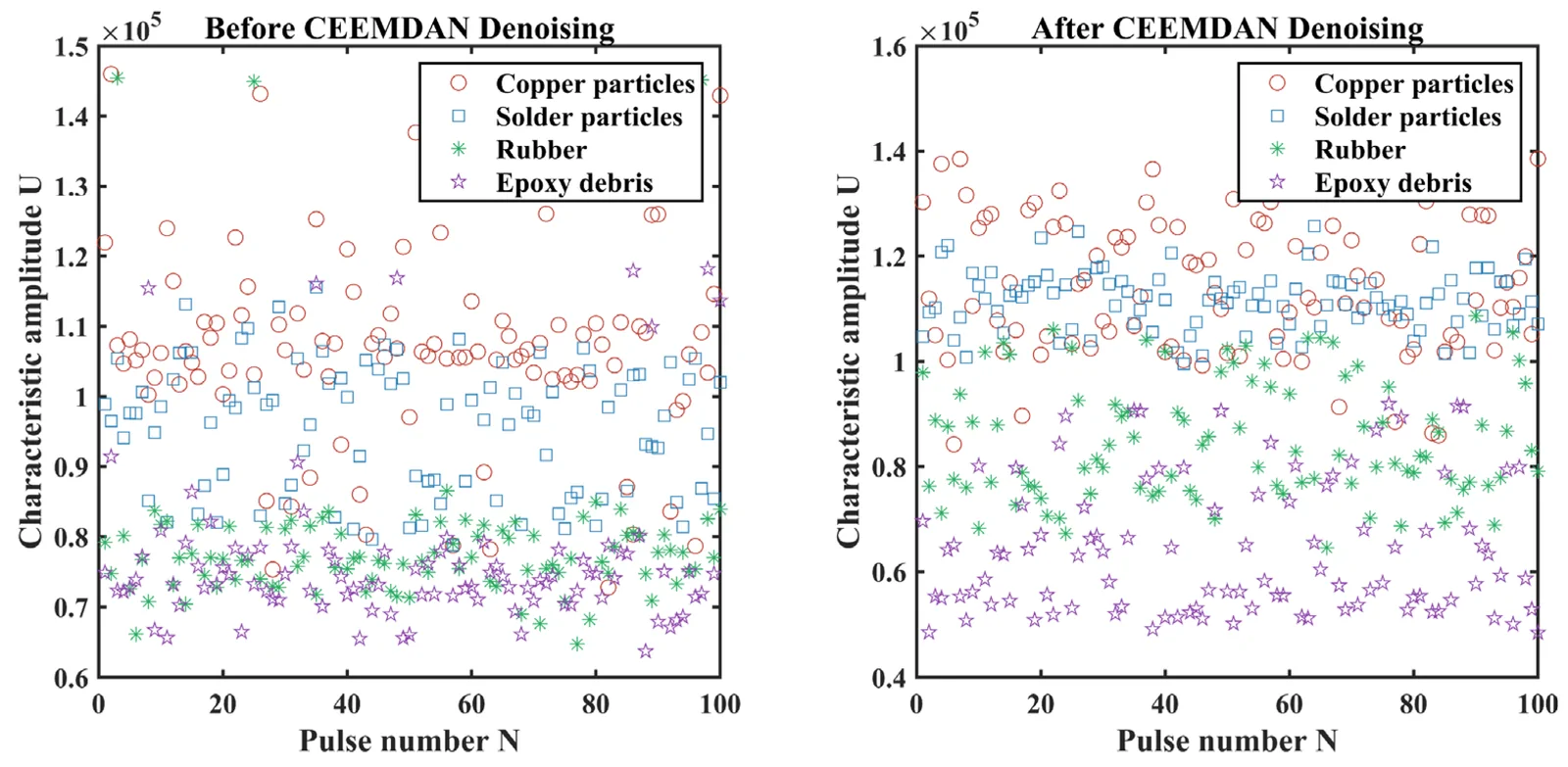
Spectral centroid before and after CEEMDAN denoising.

**Figure 11:**
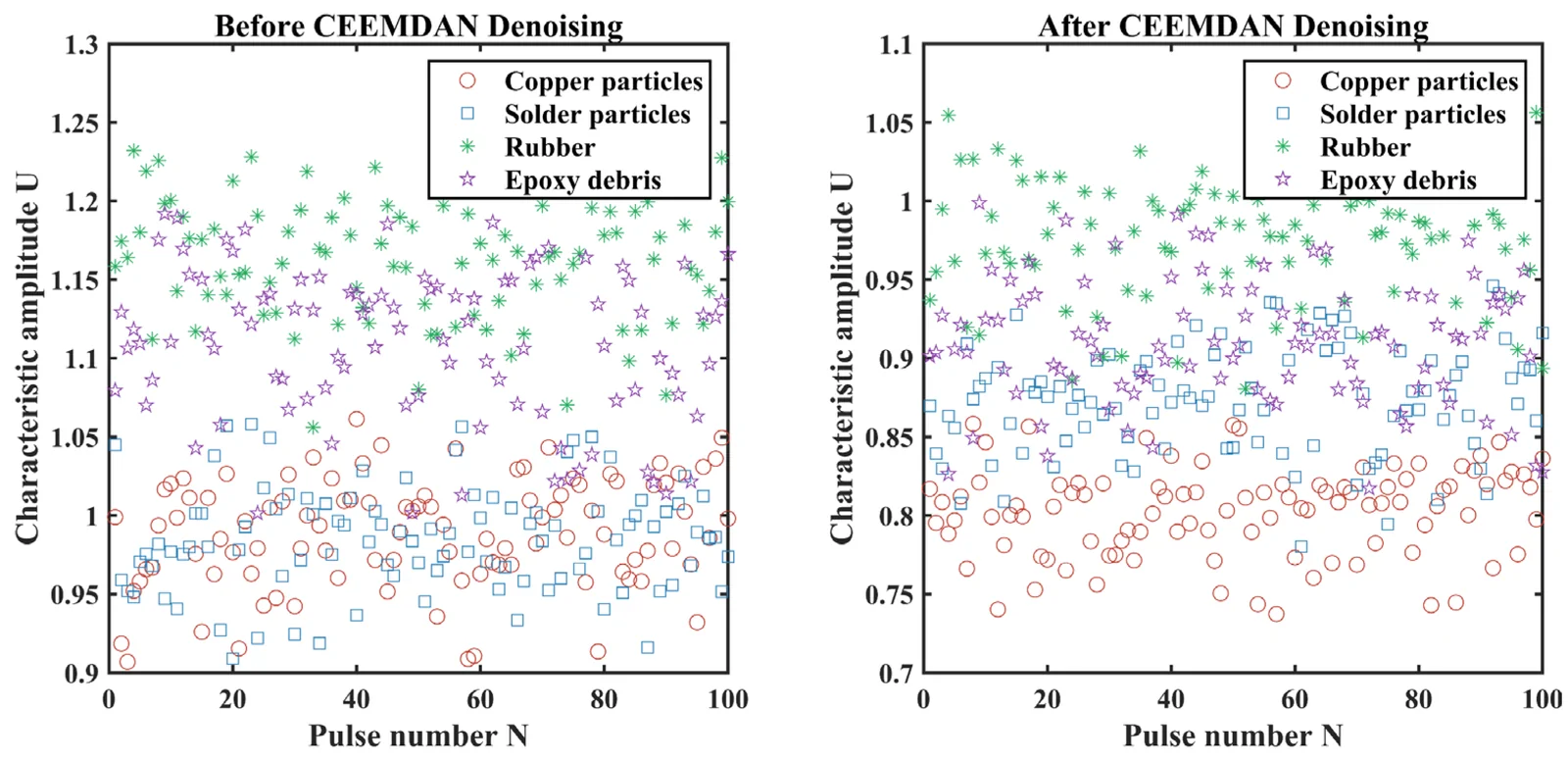
Pulse duration before and after CEEMDAN denoising.

**Figure 12:**
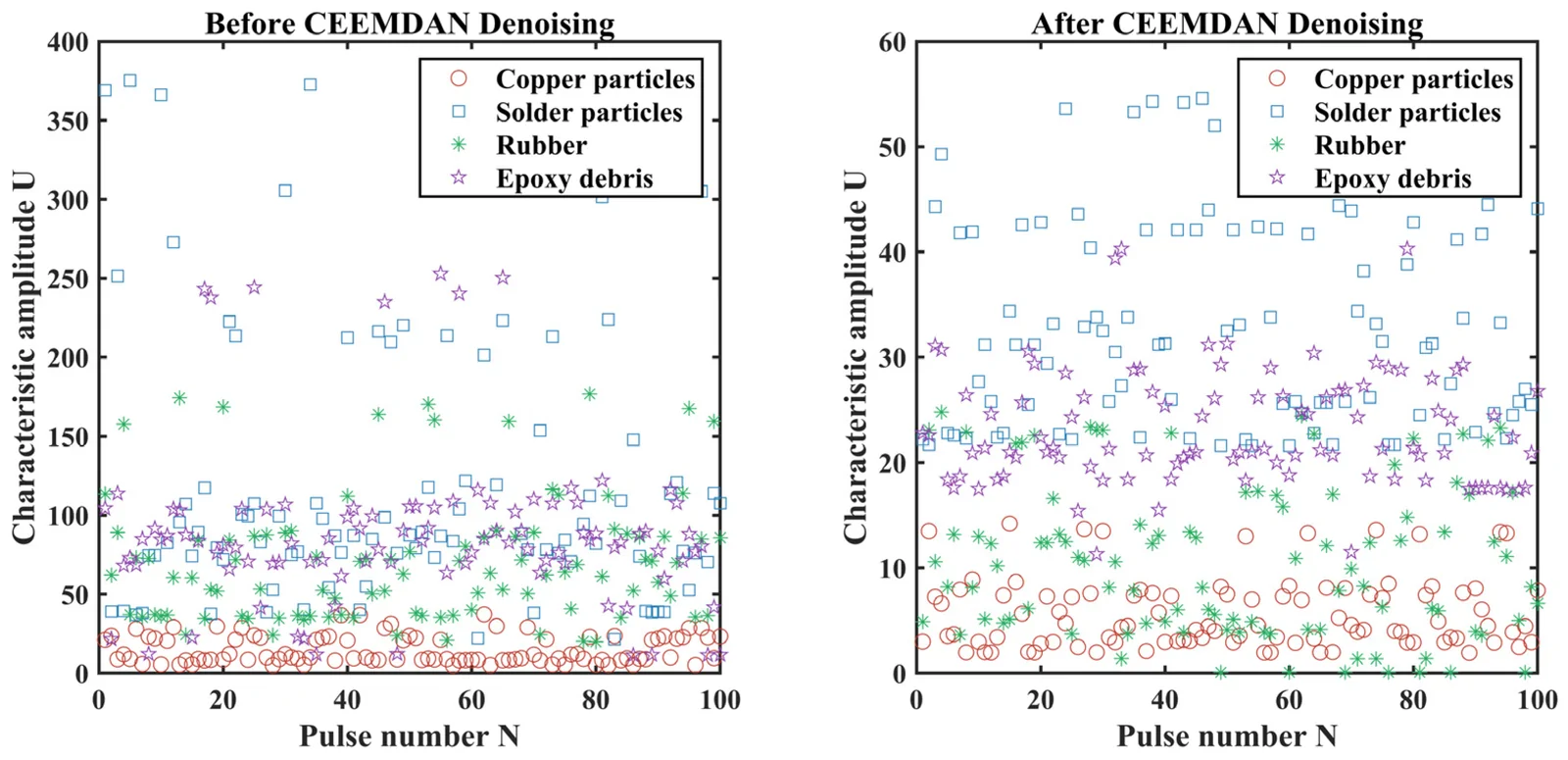
Wavelet mean-amplitude product before and after CEEMDAN denoising.

After denoising, effective features must be extracted to characterize the collision pulses for material classification. Raw PIND signals are contaminated by ambient noise and intrinsic device vibration, and direct feature extraction from raw signals leads to feature aliasing and poor discriminability. In this study, the same seven-dimensional feature set is extracted from the signals processed by all five denoising methods (no denoising, EMD, EEMD, wavelet threshold, and CEEMDAN) to ensure a fair comparison. The seven features are: time-domain energy, spectrum variance, energy–entropy ratio, autocorrelation peak, spectral centroid, pulse duration, and wavelet mean-amplitude product. These features characterize the collision pulses from complementary perspectives of time-domain intensity, frequency-domain distribution, waveform complexity, and temporal scale. The scatter plots in Figure 7, Figure 8, Figure 9, Figure 10, Figure 11 and Figure 12 present the raw feature values without normalization, as each feature has its own physical dimension and numerical range. These plots are intended to provide a qualitative visualization of the effect of CEEMDAN denoising on each feature, rather than a quantitative comparison across different features. Normalization is applied only when the features are fed into the neural network for classification.

#### 3.3.1. Time-Domain Energy

Time-domain energy is a basic time-domain statistical feature that reflects the overall signal intensity. It is calculated by integrating the squared amplitude over the entire pulse segment and characterizes the total vibration intensity of a single collision pulse [21,22,23]. In PIND inspection of sealed electronic devices, the material density, geometric size, and contact stiffness of loose particles directly determine the energy output of collision impacts. Copper and solder particles have high density and produce strong collision impacts, resulting in higher time-domain energy. In contrast, rubber and epoxy particles are softer or have smaller equivalent collision masses, producing lower-energy collision vibrations.

As shown in Figure 7, the left and right subplots present the time-domain energy distributions of four types of loose particles before and after CEEMDAN denoising. In the legend, red hollow circles represent copper particles, blue hollow squares represent solder particles, green asterisks represent rubber particles, and purple pentagrams represent epoxy particles.

Before denoising, the raw pulse signals are contaminated by background noise, and the feature points are widely scattered. The four material categories overlap substantially: solder particles occupy the upper range, rubber particles are concentrated in the lower range, and copper and epoxy particles are intermingled in the middle. After CEEMDAN reconstruction and denoising, the random background noise is removed. Solder particles shift to the upper range and form a relatively distinct cluster, while epoxy particles move to the lower range and are separated from the other three materials. Copper and rubber particles remain in the middle range with partial overlap, but their overall dispersion is reduced compared with the raw signals.

#### 3.3.2. Spectrum Variance

Spectrum variance characterizes the dispersion of frequency-domain energy distribution and quantifies the concentration of signal frequency components. In the PIND inspection of sealed devices, differences in particle material, equivalent collision mass, and contact stiffness alter the frequency-domain distribution of collision-induced vibration. Particles that produce spectra with distinct resonant peaks exhibit larger variance due to the contrast between peak and background energy, whereas particles with relatively flat spectra show smaller variance values [24].

As shown in Figure 8, the left and right subplots present the spectrum variance distributions of four types of loose particles before and after CEEMDAN denoising. Before denoising, the raw pulse signals are affected by background noise. Copper particles occupy the upper range with the largest variance values, solder particles lie in the upper-middle range with broad dispersion, epoxy particles are concentrated in the middle range, and rubber particles occupy the lower range. Copper and solder particles overlap substantially, as do rubber and epoxy particles. After CEEMDAN reconstruction and denoising, the overall dispersion of each category is reduced, and the inter-class boundaries become clearer. Copper particles remain in the upper range, solder particles shift to the upper-middle range, epoxy particles occupy the middle range, and rubber particles are concentrated in the lower-middle range. Partial overlap still exists between copper and solder particles and between rubber and epoxy particles, but the overall separability is improved compared with the raw signals.

#### 3.3.3. Energy–Entropy Ratio

The energy–entropy ratio is defined as the ratio between signal energy and spectral information entropy, which comprehensively evaluates the orderliness and concentration of signal-energy distribution. A signal with high energy and low entropy yields a large energy–entropy ratio, which corresponds to concentrated energy and a simple waveform pattern such as a single intense impact. In contrast, a signal with low energy and high entropy presents a smaller ratio, which stands for dispersed energy and complicated patterns like continuous vibration or background noise. For PIND signals, foreign-object collisions of different materials generate vibration-impact modes with varying complexity. Accordingly, the energy–entropy ratio distinguishes impulsive and vibrational components and provides an essential criterion for material classification.

This feature exhibits prominent noise-insensitive characteristics. After CEEMDAN denoising, total signal energy and spectral information entropy decrease synchronously, so their ratio barely changes. The denoising operation cannot further improve its inter-class discrimination performance. Consequently, it is merely extracted as an independent feature and incorporated into the fused-feature set. The final classification accuracy is improved by complementary effects of multiple features.

#### 3.3.4. Autocorrelation Peak

The autocorrelation-peak value characterizes the self-similarity of a signal under varying time-delay conditions. A higher peak value indicates stronger periodicity and waveform repeatability. For PIND-acquired signals, collisions between loose particles and the inner wall of sealed components generate transient-impact waveforms. The autocorrelation-peak value quantifies the self-similarity of the signal under different time delays and reflects the sharpness of the impact pulse. A larger peak corresponds to a more defined single-impulse waveform, while a lower value indicates that the waveform contains more sustained vibration and background noise components. Since particles of different materials produce impact waveforms with distinct degrees of oscillatory tailing, the autocorrelation peak indirectly reflects the contact stiffness and damping properties of the colliding particle, thereby carrying material-related information.

As shown in Figure 9, the left and right subplots present the autocorrelation peak distributions of four types of loose particles before and after CEEMDAN denoising. Before denoising, the raw signals are affected by background noise, and the feature points are widely scattered. Solder particles occupy the upper range with the largest peak values, copper particles lie in the upper-middle range with broad dispersion, epoxy particles are concentrated in the middle range, and rubber particles occupy the lower range. Substantial overlap exists between copper and solder particles. After CEEMDAN reconstruction and denoising, the random fluctuations are suppressed, and the inter-class separation is improved. Copper particles shift to the upper range, solder particles move to the middle range, rubber particles occupy the lower-middle range, and epoxy particles are concentrated at the bottom and separated from the other three materials. Partial overlap remains between copper and solder particles, but the overall discriminability is enhanced compared with the raw signals.

#### 3.3.5. Spectral Centroid

The spectral centroid represents the frequency at which the energy distribution of a signal is centered, commonly described as the “center of gravity” of the spectrum. It is calculated as the weighted mean of the frequencies present in the signal, with the magnitude of each frequency component serving as the weight. A higher spectral centroid indicates that the signal energy is concentrated in higher frequency bands, whereas a lower value indicates a dominance of low-frequency components. For PIND signals, metallic particles with high stiffness generate collision vibrations rich in high-frequency content, resulting in higher spectral centroid values, while nonmetallic particles with softer textures produce more low-frequency damped oscillations and thus lower centroid values. The spectral centroid provides frequency-domain information distinct from time-domain energy: the latter reflects overall signal intensity, while the former indicates where the spectral energy is concentrated, thereby reducing feature redundancy [25].

As shown in Figure 10, the left and right subplots present the spectral centroid distributions of four types of loose particles before and after CEEMDAN denoising. Before denoising, the raw signals are affected by background noise. Copper particles occupy the upper range with the highest centroid values, solder particles lie in the upper-middle range, rubber particles are concentrated in the lower-middle range, and epoxy particles occupy the lowest range. Copper and solder particles overlap substantially, as do rubber and epoxy particles. After CEEMDAN reconstruction and denoising, the inter-class separation is improved. Copper particles remain in the upper range, solder particles lie in the upper-middle range with partial overlap with copper, rubber particles occupy the lower-middle range, and epoxy particles are concentrated at the bottom and fully separated from the other three materials. The overall discriminability is enhanced compared with the raw signals.

#### 3.3.6. Pulse Duration (PDT)

Pulse duration represents the time span from the onset to the complete attenuation of a collision-induced signal, which characterizes the time-scale property of impact waveforms. During PIND tests, the material, mass, geometry, and collision angle of loose particles alter the attenuation behavior of impact waveforms [26,27,28]. Metallic particles produce transient pulses with rapid signal decay and short duration, whereas nonmetallic materials such as rubber and epoxy generate elastic-damped vibration with prolonged waveform tails and slower attenuation. Hence, pulse duration serves as a time-domain feature for distinguishing particle materials.

As shown in Figure 11, the left and right subplots present the pulse duration distributions of four types of loose particles before and after CEEMDAN denoising. Before denoising, the raw signals are affected by background noise, and the pulse boundaries are ambiguous. Rubber particles occupy the upper range with the longest duration, epoxy particles lie in the upper-middle range, and copper and solder particles are concentrated in the lower range with substantial overlap. After CEEMDAN reconstruction and denoising, the noise-induced jitter on waveform edges is removed, and the pulse boundaries are more precisely determined. Copper particles shift to the bottom range and are separated from the other three materials, solder particles lie in the lower-middle range, epoxy particles occupy the upper-middle range, and rubber particles remain in the upper range. The inter-class separability is improved compared with the raw signals, although partial overlap remains between epoxy and rubber particles.

#### 3.3.7. Wavelet Mean-Amplitude Product

The wavelet mean-amplitude-product feature is constructed based on wavelet decomposition of raw pulse signals. It quantifies energy distribution and transient-burst intensity on high-frequency detail components. For PIND-test pulses, high-frequency wavelet components capture transient-impact information generated from foreign-object collisions. This feature is sensitive to impact intensity and local waveform variation, which distinguishes subtle waveform differences induced by different-material collisions. The feature-extraction steps and formulas are given below [29].

**Step1:** The original collision-induced time-domain signal is segmented by windowing. The i-th frame is defined as $x_{i}(n)$,n=1,2,...,N, where N denotes frame length [30,31].

**Step2:** Each frame $x_{i}(n)$ is decomposed into 10-level discrete wavelet coefficients using the db4 wavelet function [32,33], and the detail coefficient at layer k is denoted as $d_{i,k}(n),k=1,2,\ldots ,10$.

**Step3:** The 10-level detail coefficients are divided into two groups: layers 1–5 and layers 6–10. The mean amplitude of the k-th layer wavelet coefficient is:

(5) $$A_{i,k}=\frac{1}{N}\sum_{n=1}^{N}\left|d_{i,k}(n)\right|$$

**Step4:** The maximum mean-amplitude values of the two groups are denoted as $A_{i,max1}$ and $A_{i,max2}$. The wavelet mean-amplitude product $F_{i}$ is defined as their product:

(6) $$A_{i,max1}=\max\left\{A_{i,1},A_{i,2},A_{i,3},A_{i,4},A_{i,5}\right\}$$

(7) $$A_{i,max2}=\max\left\{A_{i,6},A_{i,7},A_{i,8},A_{i,9},A_{i,10}\right\}$$

(8) $$F_{i}=A_{i,max1}\cdot A_{i,max2}$$

Following the above feature extraction pipeline, the wavelet mean-amplitude product is calculated for all pulse signals before and after CEEMDAN denoising.

As shown in Figure 12, before denoising the feature points are widely scattered, with solder particles in the upper range, epoxy and rubber particles in the middle ranges, and copper particles at the bottom. After CEEMDAN denoising, the four categories form clear hierarchical layers: solder particles remain in the upper range, epoxy particles in the upper-middle, rubber particles in the lower-middle, and copper particles at the bottom, with only minor overlap between rubber and epoxy particles.

### 3.4. BP Neural Network

To evaluate the effect of CEEMDAN decomposition on classification performance, a back-propagation (BP) neural network is used for loose particle material identification [34,35]. Five groups with identical network structures are compared, differing only in the denoising method: no denoising (control), EMD, EEMD, wavelet threshold, and CEEMDAN (proposed). Each group is repeated four times, and results are given as mean accuracy ± standard deviation.

#### 3.4.1. Network Architecture and Parameter Configuration

A two-hidden-layer BP neural network is adopted. The input layer contains seven neurons corresponding to the 7-dimensional feature vector, with Z-score standardization applied before feeding into the network. The first and second hidden layers contain 32 and 16 neurons, respectively, with tansig activation functions. The Levenberg–Marquardt algorithm is used for training, with L2 regularization (coefficient 0.0008) to mitigate overfitting. The output layer has four neurons with softmax activation, matching the four material types. The maximum training epoch is set to 2000, with an early-stopping strategy (maximum failure count of 20).

#### 3.4.2. Experimental Design and Dataset Partitioning

All five groups use the same dataset partition described in Section 2.3, with 1200 training samples from three mass gradients (0.1 mg, 0.2 mg, and 0.8 mg) and 400 test samples from the unseen 0.4 mg gradient.

Model training and evaluation: Each group is trained on its respective training set and tested on the same test set. Each experiment is repeated four times with random initializations. Results are compared in terms of overall classification accuracy, confusion matrices, and per-class recall rate.

## 4. Results

Five experimental groups are configured with identical network architectures and hyperparameters, differing only in the signal preprocessing scheme: a control group with features extracted directly from concatenated pulses, three conventional denoising groups using EMD, EEMD, and wavelet thresholding, and an experimental group using CEEMDAN-reconstructed signals. Each group is repeated four times with independent random initializations, and results are reported as mean accuracy ± standard deviation. The confusion matrices presented in this section are averaged over four independent runs, with values rounded to the nearest integer. Classification performance is evaluated through confusion matrices and classification scatter plots.

### 4.1. Confusion Matrix Analysis

In each confusion matrix, the horizontal axis represents the true class and the vertical axis represents the predicted class. Classes 1 through 4 correspond to copper particles, solder particles, rubber particles, and epoxy particles, respectively.

#### 4.1.1. Control Group

**Figure 13:**
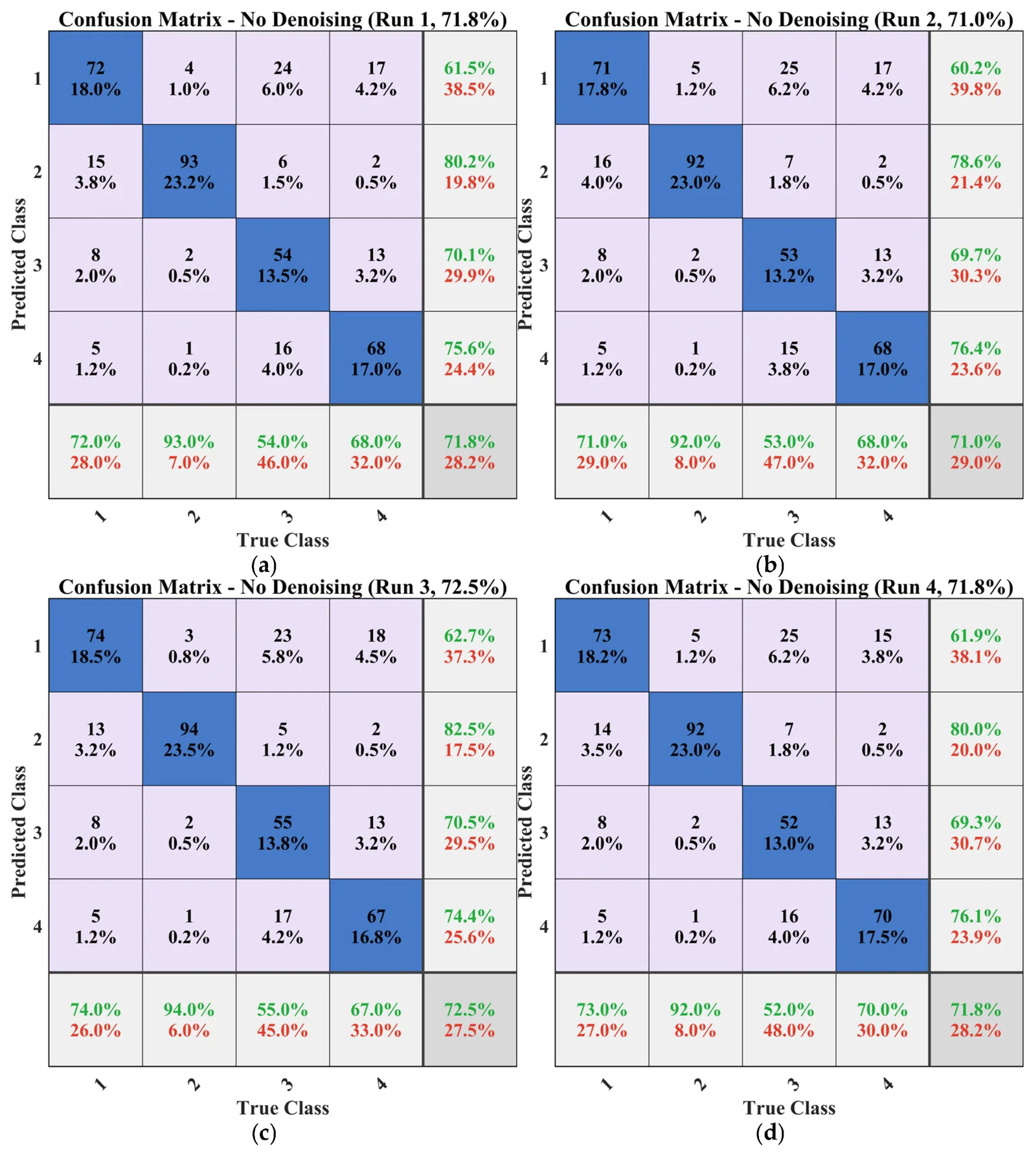
Confusion matrix of the control group (no denoising).

Figure 13 shows the confusion matrix of the control group, averaged over four independent repeated runs. The overall accuracy is 71.8 ± 0.6%.

Solder particles (Class 2) attain the highest recall rate at 92.8%, as their high-density metallic collision pulses exhibit waveform characteristics that are comparatively less sensitive to background noise. Copper particles (Class 1) reach a recall rate of 72.5%, with 14.5% of copper samples assigned to the solder class. This cross-class confusion is attributed to the shared metallic properties of copper and solder, which produce similar collision vibration patterns; background noise further obscures the inter-class differences.

The two polymer classes show lower recall rates: 53.5% for rubber particles (Class 3) and 68.3% for epoxy particles (Class 4). Among rubber samples, 24.3% are classified as copper and 15.5% as epoxy. Among epoxy samples, 17.0% are classified as copper and 13.3% as rubber. The mutual misclassification between rubber and epoxy accounts for the largest portion of classification errors and is the primary factor limiting overall accuracy.

#### 4.1.2. Conventional Denoising Methods

**Figure 14:**
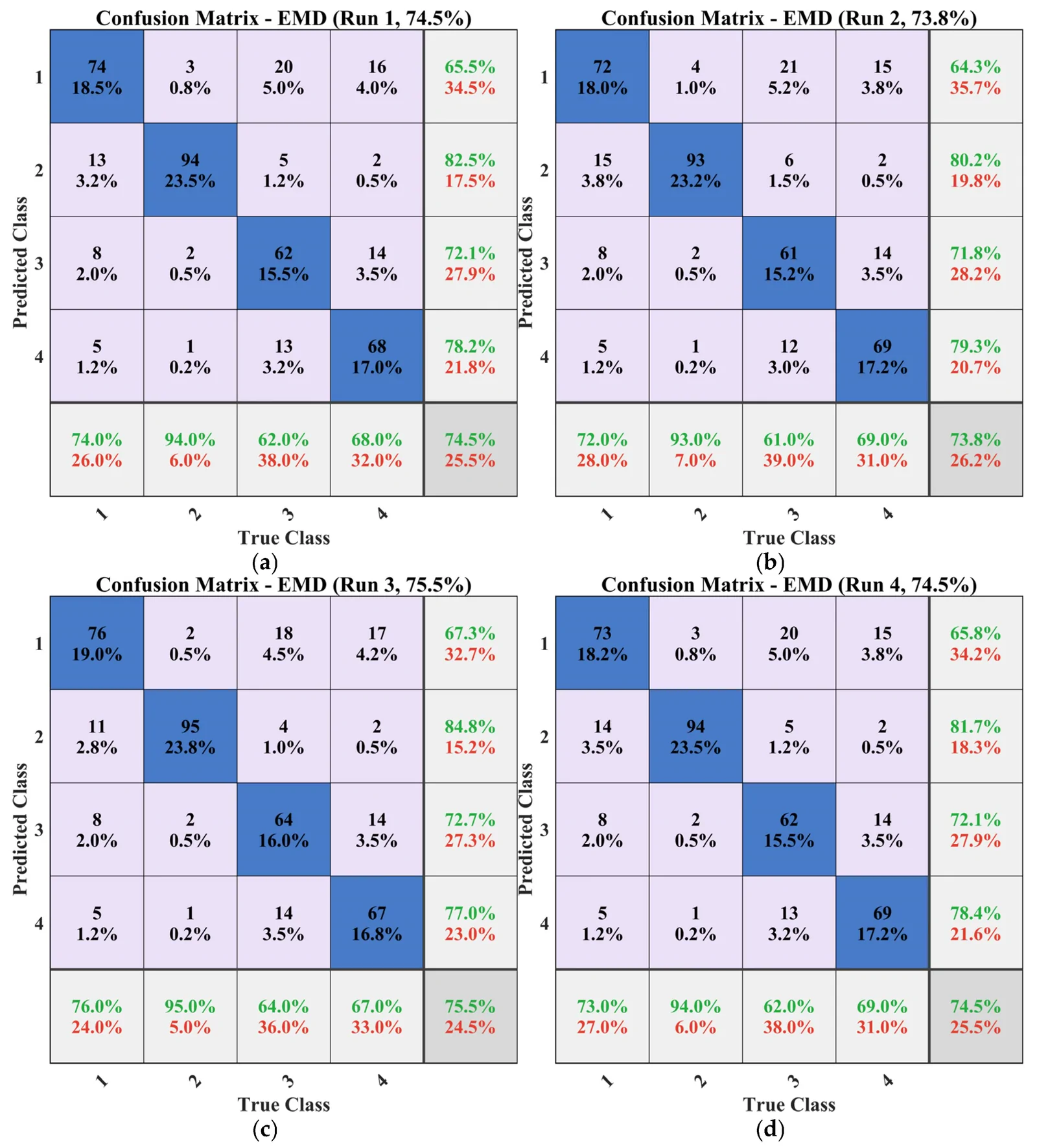
Confusion matrix of the EMD group.

**Figure 15:**
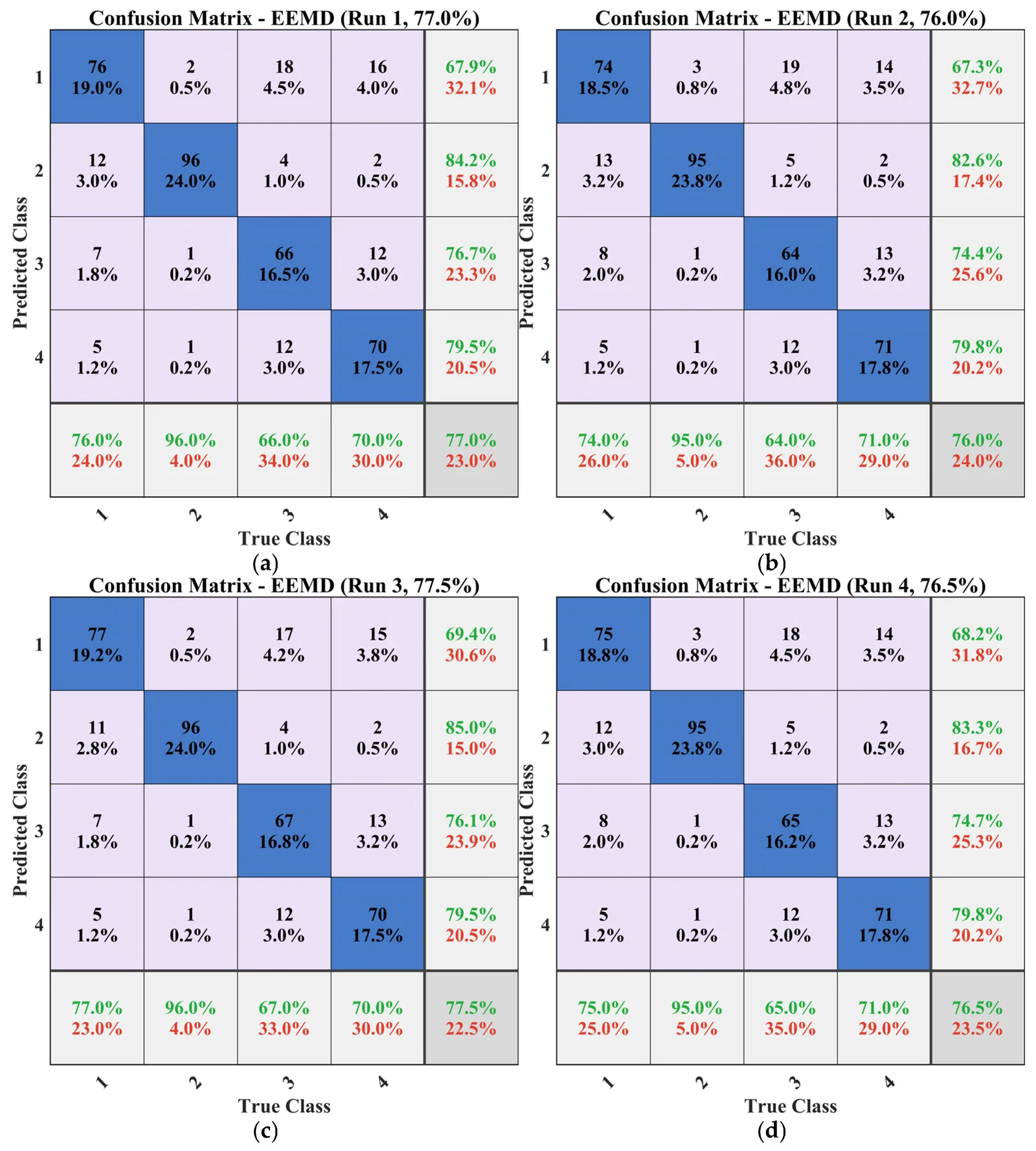
Confusion matrix of the EEMD group.

**Figure 16:**
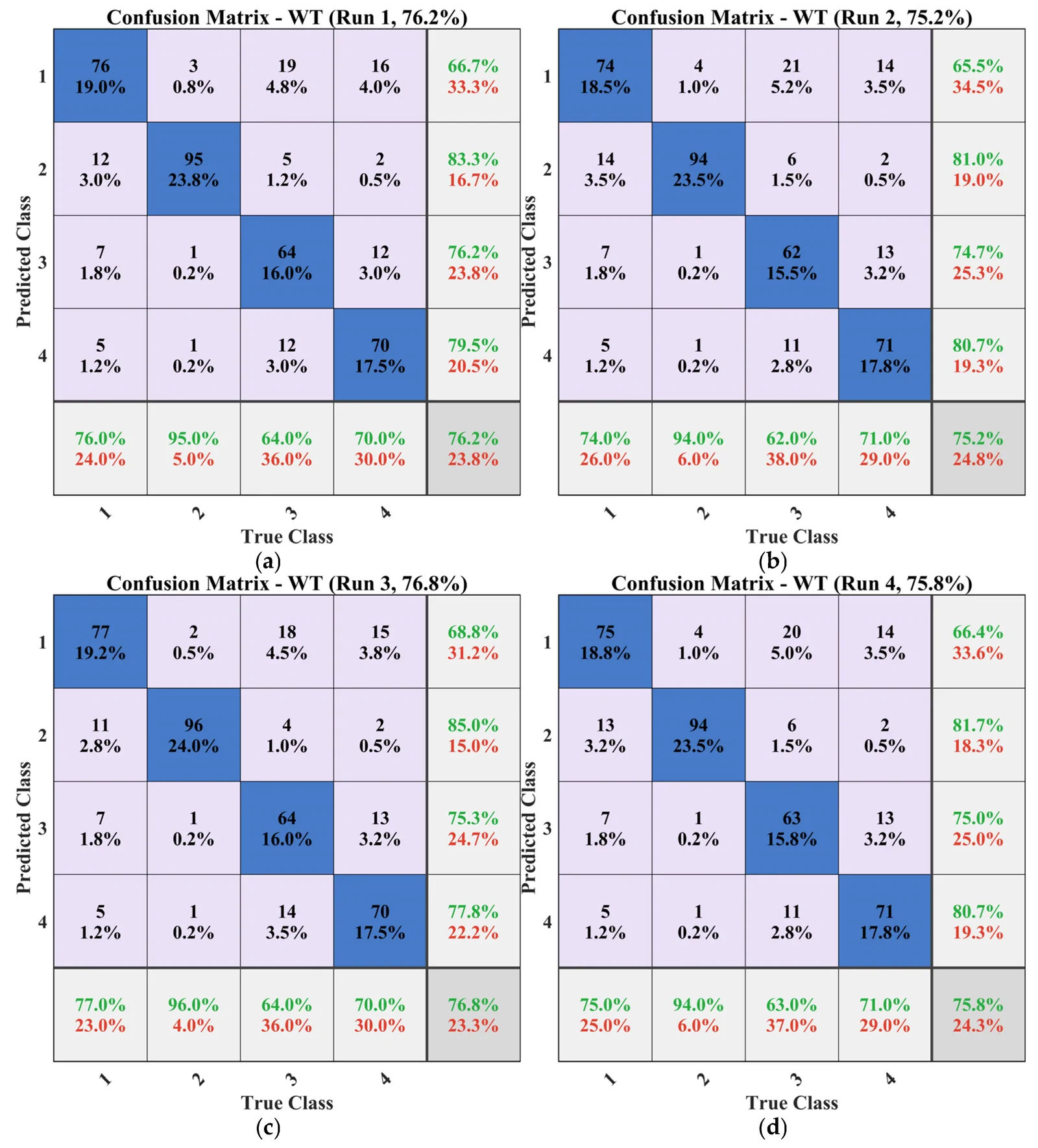
Confusion matrix of the wavelet threshold group.

Three conventional denoising methods are evaluated as benchmarks: EMD, EEMD, and wavelet thresholding. Their confusion matrices, averaged over four independent repeated runs, are shown in Figure 14, Figure 15 and Figure 16, respectively.

EMD. The EMD-based group achieves an overall accuracy of 74.5 ± 0.7%, corresponding to a 2.7 percentage point increase over the control group. Recall rates are 73.8% for copper, 94.0% for solder, 62.3% for rubber, and 68.3% for epoxy. The mode-mixing phenomenon in EMD causes collision pulse components and noise components to coexist within the same IMF, which restricts denoising performance. The largest increase is observed for rubber particles, whose recall rate rises by 8.8 percentage points; the remaining three classes show changes within 2 percentage points.

EEMD. The EEMD-based group obtains an overall accuracy of 76.8 ± 0.6%, 5.0 percentage points above the control group. Recall rates are 75.5% for copper, 95.5% for solder, 65.5% for rubber, and 70.5% for epoxy. The addition of auxiliary white noise prior to decomposition mitigates mode mixing to a certain degree, resulting in better separation between signal-dominated and noise-dominated IMFs. However, residual components from the added noise remain in the reconstructed signal, and the fixed IMF selection criterion does not adapt to material-specific differences in valid component distribution. Compared with EMD, EEMD further reduces the misclassification of copper as solder to 12.5%, and rubber recall increases by an additional 3.2 percentage points.

Wavelet Threshold. The wavelet threshold group reaches an overall accuracy of 76.0 ± 0.6%, 4.2 percentage points above the control group. Recall rates are 75.5% for copper, 94.8% for solder, 63.3% for rubber, and 70.5% for epoxy. The sqtwolog threshold with soft thresholding is applied to db4 wavelet coefficients at five decomposition levels. This configuration suppresses high-frequency Gaussian noise, but the fixed threshold does not accommodate the non-stationary and impulsive nature of PIND signals. Low-amplitude collision details in polymer pulses are removed together with noise, which limits the gain in rubber and epoxy classification.

Among the three conventional methods, EEMD yields the highest overall accuracy, followed by wavelet thresholding and EMD. All three methods show limited improvement for the two polymer classes, and mutual misclassification between rubber and epoxy remains present.

#### 4.1.3. CEEMDAN (Proposed Method)

**Figure 17:**
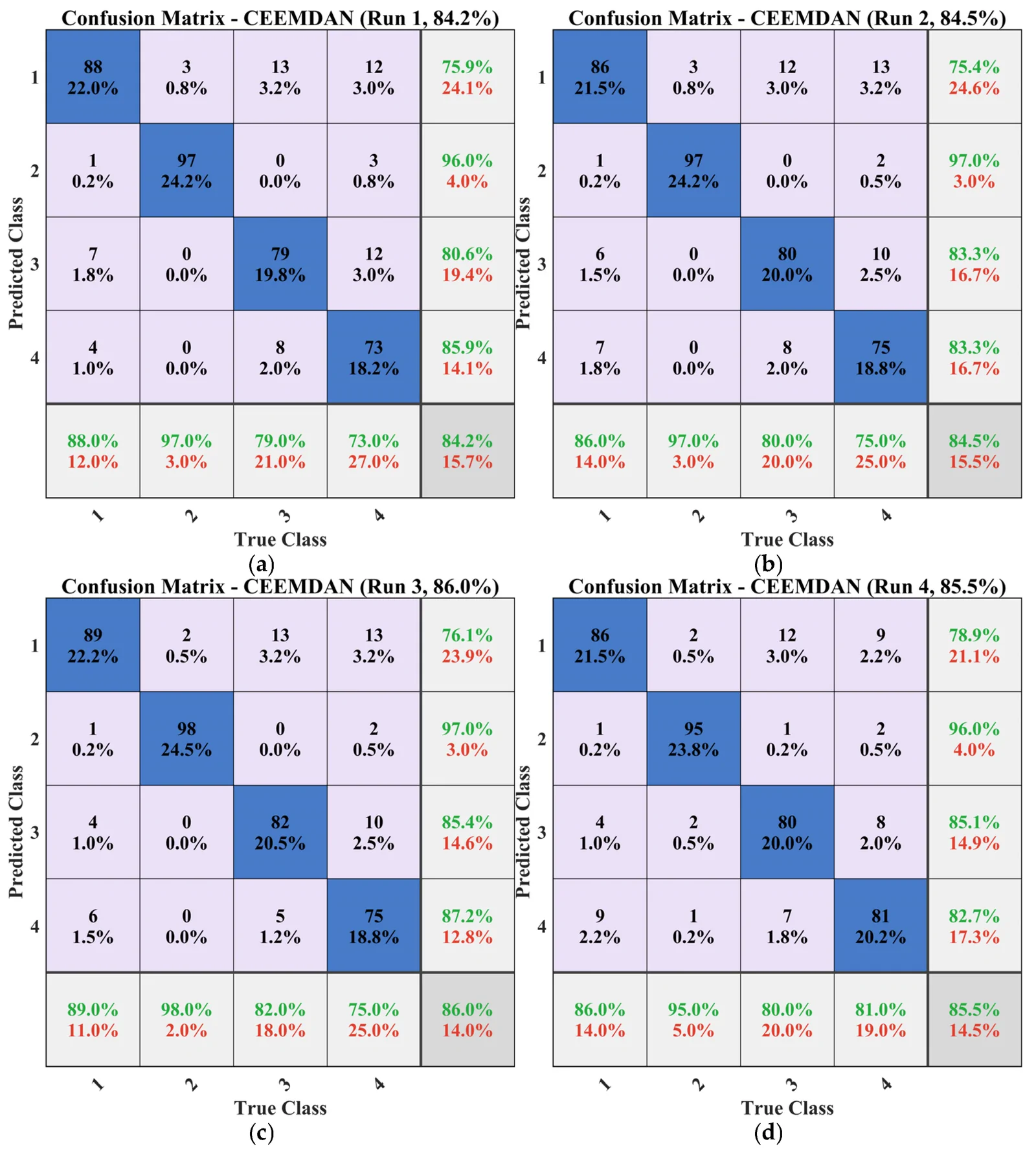
Confusion matrix of the CEEMDAN group.

Figure 17 shows the confusion matrix of the CEEMDAN group, averaged over four independent repeated runs. The overall accuracy is 85.1 ± 0.8%, which is 13.3 percentage points higher than the control group and 8.3 percentage points higher than EEMD, the best-performing conventional method.

Recall rates increase across all four classes. Solder particles remain at 96.0%, and copper particles rise from 72.5% to 85.0%. The misclassification of copper as solder decreases from 14.5% to 8.0%. For the two polymer classes, rubber recall increases from 53.5% to 77.0%, and epoxy recall increases from 68.3% to 82.0%. Rubber misclassified as epoxy decreases from 15.5% to 14.0%, and epoxy misclassified as rubber decreases from 13.3% to 11.0%. Misclassification of polymer particles as copper decreases from 24.3% to 6.0% for rubber and from 17.0% to 5.0% for epoxy.

The kurtosis-based IMF screening removes high-frequency impulsive noise components (IMF1–IMF2) while retaining collision pulse structures (IMF3–IMF7). This selection preserves waveform differences between metallic and polymer particles that are otherwise obscured by noise.

### 4.2. Visualization Analysis of Sample Classification Scatter Plots

Classification scatter plots are generated for the control group and the CEEMDAN group to provide an intuitive view of sample-level classification results. The horizontal axis is the sample index, and the vertical axis is the predicted class output by the BP neural network. Blue markers denote correctly classified samples where the predicted label matches the true label, and red markers denote misclassified samples. Each class comprises 100 consecutive test samples, ordered from bottom to top as copper particles, solder particles, rubber particles, and epoxy particles.

#### 4.2.1. Control Group

**Figure 18:**
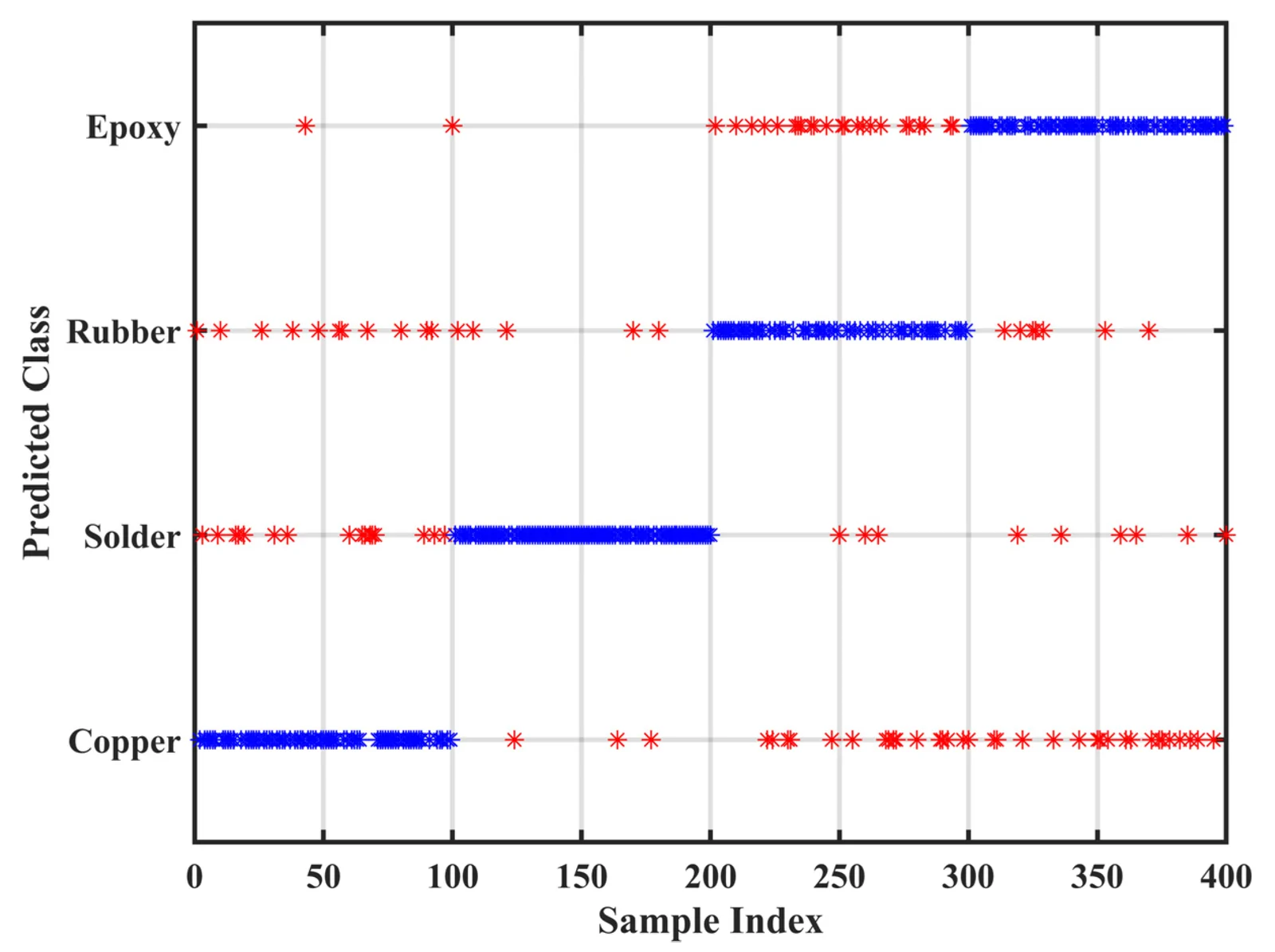
Classification Scatter Plot of the Control Group (No Denoising).

Figure 18 shows the classification scatter plot of the control group. Misclassified samples appear in all four class intervals.

Within the copper interval, most samples in the first half are correctly classified, while the density of red markers increases in the second half, with some samples extending into the solder interval above. In the solder interval, misclassifications scatter near both ends, with a few beyond the interval boundaries. The rubber and epoxy intervals have the densest red markers. In the rubber interval, misclassifications concentrate at the beginning, with samples assigned to copper (below) and epoxy (above). In the epoxy interval, red markers appear at both ends, with many samples assigned to rubber. This distribution is consistent with the confusion matrix results in Section 4.1.1: feature distributions of different materials overlap under background noise, particularly at class boundaries.

#### 4.2.2. Experimental Group

**Figure 19:**
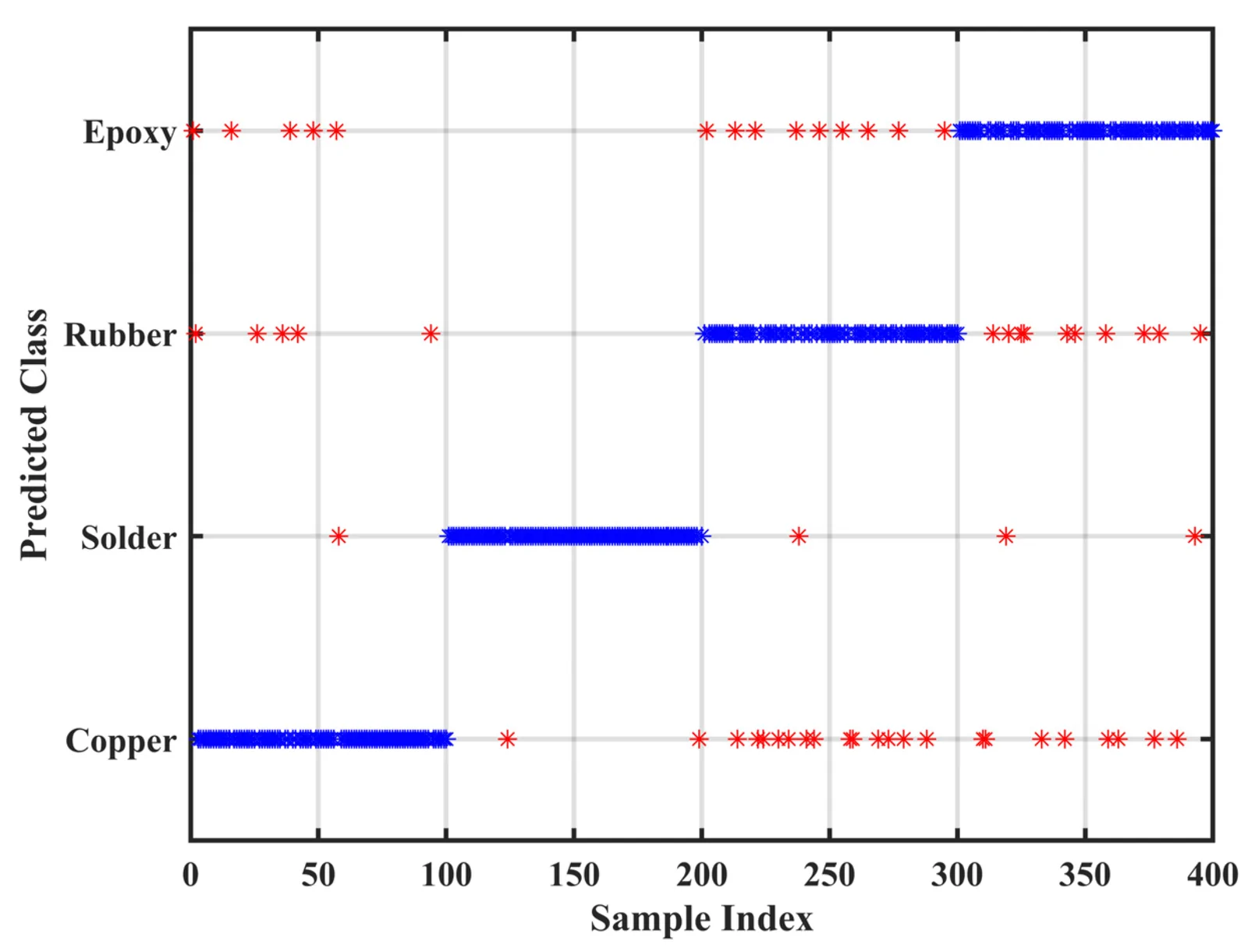
Classification scatter plot of the experimental group (CEEMDAN).

Figure 19 shows the classification scatter plot of the CEEMDAN group. The number of misclassified samples decreases in all four intervals.

In the solder interval, nearly all samples are correctly classified, with isolated red markers only at the interval boundaries. The copper interval also shows fewer misclassifications, with the correctly classified region extending further toward the end of the interval. In the rubber and epoxy intervals, red markers remain at the beginning and end of each interval, but their count is lower than in the control group. Correctly classified samples form continuous distributions within their respective intervals, and the boundaries between adjacent classes are more distinct.

These observations are consistent with the confusion matrix analysis in Section 4.1. Remaining misclassifications concentrate at the transitions between adjacent classes, where feature distributions still overlap to some extent.

## 5. Discussion

Across the four repeated experiments, CEEMDAN decomposition and reconstruction raise the overall material identification accuracy from 71.8 ± 0.6% (control group, no denoising) to 85.1 ± 0.8%. This corresponds to a 13.3 percentage point improvement over the control group and an 8.3 percentage point improvement over EEMD (76.8 ± 0.6%), the best-performing conventional method among the three benchmarks. EMD (74.5 ± 0.7%), wavelet thresholding (76.0 ± 0.6%) and EEMD all yield moderate gains over the control group but remain below CEEMDAN. Their limited improvement is attributed to mode mixing in EMD, residual auxiliary noise in EEMD, and fixed-threshold inflexibility in wavelet denoising, each of which restricts the preservation of low-amplitude collision details in polymer particle pulses.

The accuracy gain is unevenly distributed across the four materials. Rubber particles benefit most, with recall increasing from 53.5% to 77.0%, followed by epoxy particles (68.3% to 82.0%) and copper particles (72.5% to 85.0%). Solder particles show the smallest gain, from 92.8% to 96.0%, as their collision pulses are already well-separated from the other materials in the raw feature space. This ordering is consistent with the underlying signal physics. Rubber and epoxy are low-energy polymer materials whose collision amplitudes lie close to the background noise floor, so residual noise distorts their features most severely and denoising yields the largest correction. Copper particles also show substantial improvement because their dominant error is misclassification as solder, and CEEMDAN reconstruction restores the frequency and time-scale differences that separate the two metals. Solder particles produce stable high-amplitude pulses with clear feature boundaries, leaving little room for further improvement.

The confusion matrices reveal two principal sources of classification error. The first is confusion between the two metals, copper and solder. In the control group, 14.5% of copper samples are predicted as solder, whereas only about 4% of solder samples are predicted as copper. This asymmetry arises because solder, as a low-melting-point soft metal, occupies a compact feature region with intermediate energy and frequency content, whereas copper has a broader distribution that overlaps with solder in certain dimensions. After CEEMDAN denoising, the copper-to-solder error decreases to 8.0%, as the reconstructed signals recover the frequency and time-scale distinctions between the two metals. Solder particles consistently achieve higher recall than copper particles in both groups for the same reason: their compact feature region is separated from both the hard metal (copper) and the two polymers, with only a small overlap with copper in high-frequency characteristics. The second principal error source is mutual confusion between the two polymers, rubber and epoxy. In the control group, 15.5% of rubber samples are classified as epoxy and 13.3% of epoxy samples as rubber, since both materials generate low-energy damped oscillations with similar frequency content and pulse duration. Denoising reduces these mutual errors to 14.0% and 11.0%, respectively. In addition, 24.3% of rubber samples and 17.0% of epoxy samples are misclassified as copper in the control group. Copper has a relatively wide feature distribution, and when noise obscures the distinguishing features of low-energy samples, the model tends to assign them to the copper category. CEEMDAN denoising lowers this error to 6.0% for rubber and 5.0% for epoxy.

The seven features used in this study capture different physical properties of the collision signals. Time-domain energy and pulse duration describe the intensity and time scale of each impact. Spectrum variance and spectral centroid characterize the frequency-domain distribution in terms of dispersion and center of gravity. The wavelet mean-amplitude product isolates transient high-frequency components through wavelet decomposition. The energy–entropy ratio and autocorrelation peak reflect waveform complexity and self-similarity. No single feature fully separates the four materials, but their combination provides a more complete representation of the collision signals. The spectral centroid is particularly informative because it conveys frequency-domain information that is not redundant with time-domain energy: the latter measures overall signal intensity, whereas the former indicates where the spectral energy is concentrated.

Most existing PIND material identification studies either extract features directly from raw signals without denoising or employ conventional denoising methods such as EMD, EEMD, or wavelet thresholding. The CEEMDAN-based preprocessing used here suppresses residual noise more effectively than these conventional approaches and improves feature separability, especially for low-energy polymer particles. However, it should be acknowledged that CEEMDAN-based denoising may still have certain limitations in residual noise suppression, as noted in recent acoustic signal processing studies [36]. Furthermore, most PIND material identification studies employ random train–test splitting, which may place samples from the same specimen or mass gradient into both training and test sets, yielding an overly optimistic estimate of generalization. The leave-one-mass-gradient-out strategy adopted in this study ensures that the test set contains a mass gradient entirely unseen during training, providing a more rigorous assessment of the model’s generalization beyond the training masses. We note that this design reduces the risk of mass-dependent overfitting but does not fully exclude the influence of particle mass on classification.

Several limitations of this work should be noted. First, the experiments are conducted in a standardized aluminum-alloy-simulated cavity, and the applicability of the proposed method to actual sealed electronic devices in industrial quality control scenarios [37] requires further verification. Second, the dataset covers four material types and four mass gradients; performance on other contaminant types and wider mass ranges remains untested. Third, all experiments are performed under controlled laboratory conditions with fixed vibration parameters and sensor placement. Finally, the BP neural network used in this work is a relatively simple classifier, and more advanced models may further improve classification performance.

## 6. Conclusions

To address the problems of background noise contamination, feature aliasing, and low recognition accuracy for polymer particles in PIND testing of sealed electronic devices, this paper proposes a loose particle material identification method combining frequency-domain variance dual-threshold endpoint detection and CEEMDAN-based feature optimization. The method integrates pulse segmentation, noise reduction through signal reconstruction, and multi-feature fusion to classify four typical loose particle materials: copper, solder, rubber, and epoxy. The main findings are summarized as follows.

(1) The frequency-domain variance dual-threshold endpoint detection algorithm effectively preprocesses long-duration noisy PIND signals. It locates and extracts valid collision pulses while removing blank noise segments and invalid baseline components, which improves the signal-to-noise ratio of the raw signals and provides clean pulse samples for subsequent feature extraction and model classification. This preprocessing step also reduces the computational cost of CEEMDAN decomposition by restricting the decomposition to concatenated valid pulse sequences rather than the full raw acquisition.

(2) The CEEMDAN decomposition combined with kurtosis-based IMF screening suppresses residual noise while retaining the waveform characteristics of collision pulses. IMFs with kurtosis above 50 (IMF1 and IMF2, dominated by impulsive noise) and the low-frequency trend term (IMF8) are discarded, while IMF3–IMF7 are retained for signal reconstruction. This approach reduces the distortion of low-energy polymer pulses from rubber and epoxy particles and improves the separability of features across different materials.

(3) Quantitative analysis of confusion matrices demonstrates the effectiveness of the proposed method. Using a leave-one-mass-gradient-out strategy with 1200 training samples and 400 test samples, and repeating each experiment four times with random initializations, five preprocessing schemes are compared: no denoising (control), EMD, EEMD, wavelet thresholding, and CEEMDAN. The overall recognition accuracy is 71.8 ± 0.6% for the control group, 74.5 ± 0.7% for EMD, 76.0 ± 0.6% for wavelet thresholding, 76.8 ± 0.6% for EEMD, and 85.1 ± 0.8% for CEEMDAN. The proposed method outperforms the best conventional benchmark (EEMD) by 8.3 percentage points. Among the four materials, rubber particles show the largest improvement, with recall increasing from 53.5% to 77.0%, followed by epoxy particles from 68.3% to 82.0% and copper particles from 72.5% to 85.0%. Solder particles, which already achieve high recall with raw features, improved from 92.8% to 96.0%. The principal classification errors—mutual confusion between copper and solder, and between rubber and epoxy—are reduced after CEEMDAN denoising.

The proposed method improves the recognition accuracy and stability of loose particle material identification under noisy conditions and provides a reference for signal processing in PIND testing of sealed electronic devices. The signal processing framework, including pulse endpoint detection, CEEMDAN denoising, and multi-feature fusion, is potentially adaptable to other cavity types with parameter recalibration, although only one standardized aluminum cavity has been experimentally investigated in this study.

Future work will focus on validating the proposed method on actual sealed electronic devices, expanding the dataset to include more material types and mass gradients, and investigating the robustness of the approach under varying testing conditions. More advanced classification models such as convolutional neural networks or ensemble methods may also be explored to further improve recognition accuracy and generalization.

## Figures and Tables

**Table 1:** Mass gradients of four types of loose particle samples.

Material	Weight 1	Weight 2	Weight 3	Weight 4
Copper particles	0.11 mg	0.20 mg	0.41 mg	0.82 mg
Solder particles	0.10 mg	0.22 mg	0.42 mg	0.80 mg
Rubber particles	0.11 mg	0.20 mg	0.40 mg	0.80 mg
Epoxy particles	0.10 mg	0.20 mg	0.40 mg	0.80 mg

**Table 2:** Kurtosis values of the eight IMF components.

IMF Component	Kurtosis	Classification	Retained
IMF1	189.12	Impulsive noise	No
IMF2	155.48	Impulsive noise	No
IMF3	22.73	Collision pulse	Yes
IMF4	34.07	Collision pulse	Yes
IMF5	23.51	Collision pulse	Yes
IMF6	10.47	Collision pulse	Yes
IMF7	9.69	Collision pulse	Yes
IMF8	3.29	Trend term	No

## Data Availability

Some or all data, models, or code that support the findings of this study are available from the corresponding author upon reasonable request.
